# Response Surface Methodology for Compressive Strength of Nano-Modified Concrete at High Temperatures

**DOI:** 10.3390/nano16201330

**Published:** 2026-10-21

**Authors:** Alaa A. Mahmoud, Hany A. Dahish, Mohammed K. Alkharisi, Mohamed A. Abouelnour, Marwa A. Sadawy, Islam N. Fathy

**Affiliations:** 1 Construction and Building Engineering Department, October High Institute for Engineering & Technology, Giza 12566, Egypt; aa1607@fayoum.edu.eg (A.A.M.);; 2 Department of Civil Engineering, College of Engineering, Qassim University, Buraidah 52571, Saudi Arabia; ha.dahish@qu.edu.sa; 3 Civil Engineering Department, Faculty of Engineering, Fayoum University, Fayoum 63514, Egypt

**Keywords:** response surface methodology, nanoparticles, mechanical properties, high temperatures

## Abstract

This study utilizes response surface methodology (RSM) to examine the effectiveness of several nanomaterials on the residual compressive strength (CS) of concrete exposed to high temperature. Three nanoparticles were used as partial cement replacement by weight at replacement ratios up to 5%: nano-granodiorite (NG), nano-magnetite (NM), and nano-lead oxide (NL). The specimens were exposed to temperatures of 400 °C, 600 °C, and 800 °C, and the residual compressive strength was determined. The experimental results exhibited that all nanoparticles in concrete specimens reduced the loss of strength at high temperature. The control specimen lost 48.39% of its compressive strength at 800 °C. A dose of 4% NG and NM resulted in an increase in residual strength of 37.87% and 22.06%, respectively, while 3% NL specimens resulted in 11.4% improvement. A regression model for predicting concrete CS was developed using RSM. The model was successful in capturing the high correlation between the input variables (temperature, type of nanomaterial, and nanoparticle dose) and the output of CS. A good agreement between model predictions and the experimental data was obtained. The results of CS optimization showed that 5% NG at 126 °C resulted in the highest compressive strength of 61.80 MPa, confirming its superiority over NM and NL for the formulation of concrete under thermal conditions. The results reveal that the utilized nanoparticles, especially NG, have great potential to be used as cementitious materials to improve the high-temperature resilience of concrete.

## 1. Introduction

Concrete is the paramount construction material in the building sector, owing to its superior attributes such as excellent workability, cost-effectiveness, high capacity to sustain mechanical loads (especially compressive stresses), durability, and resistance to aggressive environments. Concrete is the most widely used human-made construction material, with global annual production estimated at approximately 25–30 billion tons [1]. Beyond its material consumption, the construction sector represents a major component of the global economy, accounting for approximately 9–13% of global gross domestic product (GDP) and employing a substantial share of the global workforce [2]. However, the scale of this activity also carries considerable environmental implications. Cement and concrete production is responsible for more than 7% of global greenhouse gas emissions, while the extraction and processing of raw materials place substantial pressure on natural resources [3].These figures underscore the importance of developing durable and resource-efficient concrete technologies that can maintain their structural performance under demanding service conditions, including exposure to fire and elevated temperatures. Exposure to such severe service conditions is highly detrimental to concrete structural integrity, significantly compromising its strength and functional capacity [4,5]. Exposure to high temperatures or fire conditions is among the most severe environmental factors that adversely affect the performance of concrete, as it induces complex physical and chemical transformations within its internal structure [6,7]. As temperature rises, both free and bound water in the pore system evaporate, leading to increased internal vapor pressure and the formation of microcracks due to non-uniform thermal expansion between the cement paste and the aggregates. At elevated temperatures, key hydration products such as calcium hydroxide (Ca(OH)_2_) and calcium silicate hydrate (C–S–H gel) begin to decompose, resulting in the loss of internal cohesion and a significant reduction in mechanical strength [8]. Moreover, the mismatch in thermal expansion coefficients between the aggregates and the cement matrix generates additional internal stresses, accelerating cracking and structural disintegration [9]. Furthermore, the dehydration and decomposition of cement minerals cause pronounced structural alterations in the cement matrix, increasing its porosity and friability. The transformation of calcium hydroxide into calcium oxide further intensifies microcrack formation, while gases produced by chemical reactions at temperatures exceeding 550 °C can create interconnected and longitudinal cracks within the binder network, drastically lowering its load-bearing capacity [10]. Another critical consequence of high-temperature exposure is spalling, which results from the synergistic action of increased internal water vapor pressure and high differential thermal stresses [11]. As the temperature reaches 800 °C, the compressive strength may drop to nearly half of its original value, accompanied by more than 10% mass loss [12]. At even higher temperatures, typically between 1000 and 1200 °C, concrete experiences severe degradation characterized by substantial mass loss and a drastic reduction in mechanical integrity, ultimately compromising its structural performance and durability [13]. Consequently, numerous codes of practice have recently evolved to mandate specific concrete design stipulations. This initiative aims to guarantee that concrete satisfactorily maintains its critical performance characteristics under exposure to elevated temperatures [14].

To enhance the ability of concrete to withstand the detrimental effects of elevated temperatures on its physical, mechanical, and durability properties, several modification strategies can be proposed. These include the incorporation of silica fume [15], fly ash [16,17], ground granulated blast furnace slag [18], glass powder [19], rice husk ash [20], and metakaolin [21] as supplementary cementitious materials (SCMs), which improve microstructural stability and reduce porosity under thermal stress. The use of heat-resistant aggregates, such as basalt, magnetite, granite, or serpentine, also helps improve thermal stability and minimize differential thermal expansion between the aggregate and the cement matrix [22,23,24]. Furthermore, the inclusion of fibers such as polypropylene, steel, or basalt fibers into concrete mixes has been shown to effectively control crack propagation, enhance ductility, and alleviate internal vapor pressure during heating [25,26,27]. Other approaches, such as the addition of pozzolanic ashes derived from agricultural waste, such as basil plant ash [28] and sugarcane bagasse ash [29], have also demonstrated potential in refining the pore structure, enhancing thermal compatibility, and improving the residual strength of concrete after exposure to elevated temperatures.

Nanomaterials stand out among various mitigation techniques due to their proven capability to significantly enhance the thermal resistance and overall performance of concrete when exposed to elevated temperatures [30]. Nanotechnology involves the manipulation and utilization of materials with particle sizes typically ranging between 1 and 100 nanometers, where unique physicochemical properties emerge due to the extremely high surface area-to-volume ratio and nanoscale reactivity [31]. When incorporated into cementitious systems, nanomaterials such as nano-silica (SiO_2_) [32], nano-alumina (Al_2_O_3_) [33], nano-titania (TiO_2_) [34], nano glass [35], nano-clay [36], and carbon-based nanomaterials (e.g., graphene oxide and carbon nanotubes) [37,38] have shown remarkable potential in improving microstructural compactness and thermal stability. The enhancement mechanisms are primarily attributed to the nanomaterials’ ability to fill micro- and nanopores, microstructural densification, enhancement of the interfacial transition zone, suppression of microcrack propagation, ability to refine the pore size distribution, and acceleration of the pozzolanic reaction leading to the formation of additional C–S–H gel, which collectively contribute to improved residual mechanical properties after heating [30,39]. In particular, nanosilica has frequently demonstrated favorable effects on residual compressive strength and spalling resistance owing to its pore-filling capacity and pozzolanic reaction, which promotes additional calcium silicate hydrate (C–S–H) formation [40,41]. Carbon-based nanomaterials, including carbon nanotubes (CNTs), graphene oxide (GO), and graphene nanoribbons, can bridge microcracks, facilitate stress transfer, and refine the pore structure, thus helping preserve mechanical integrity after fire exposure [38,42]. Furthermore, certain nanomaterials may mitigate explosive spalling by facilitating vapor-pressure dissipation and reducing internal damage caused by moisture migration during heating [43]. Nano clay and nano alumina can also enhance thermal resistance by refining the matrix and reducing crack development, although their effectiveness depends on dosage and dispersion quality [44,45]. Moreover, optimized hybrid systems combining nanomaterials with supplementary cementitious materials or fibers may provide greater benefits than individual additives [46,47]. Improvements in residual strength have been reported following exposure to temperatures ranging from 200 to 800 °C and, in some cases, up to 1000 °C, depending on the material composition and mixture design [46,48]. Nevertheless, these improvements are not universal. At extreme temperatures, particularly around 800–925 °C, accumulated vapor pressure, extensive microcracking, and thermal degradation may outweigh the benefits of matrix densification, resulting in strength loss even in nano-modified concrete [49,50]. Therefore, the high-temperature performance of nano-modified concrete depends strongly on nanomaterial type, dosage, dispersion, aggregate characteristics, and exposure severity.

Three different types of nanomaterials, specifically nano granodiorite (NG), nano magnetite (NM), and nano lead oxide (NL), were incorporated in this study to examine their effect on the compressive strength of concrete subjected to elevated temperatures. NL is a rarely used additive in concrete; its ultra-fine particle size contributes to filling micro voids within the cement matrix and improving interfacial bonding between the hydration products [51]. This densification of the microstructure not only reduces porosity but also can enhance concrete’s resistance to thermal degradation. Furthermore, previous studies have demonstrated the effectiveness of NL in enhancing the gamma-ray attenuation capacity of concrete under conventional conditions and aggressive exposure environments, including elevated temperatures exposure [51,52]. These findings may provide a positive indication of the potential of NL to fill pores within the concrete matrix and improve its mechanical resistance to high-temperature exposure.

NM, which is a magnetic nanoparticle of iron oxide with high thermal and chemical stability, has shown promise in enhancing both the mechanical and durability characteristics of cement-based composites [53]. It acts as a nucleation aid, refines the microstructure, and reduces porosity/cracks; optimal doses are typically around 0.25–3 wt.% depending on mix design [54]. Another study highlighted that Fe_3_O_4_ nanoparticles decrease porosity, reduce connecting paths of the pore structure, and improve resistance to chloride (Cl-) attack [55]. NM’s fine particle size allows it to act as a micro-filler, reducing pore connectivity, while its high thermal conductivity facilitates uniform heat distribution within the matrix, minimizing localized thermal stresses. These combined effects are expected to mitigate cracking and loss of strength under high-temperature conditions, making NM a suitable candidate for thermally resistant concrete formulations. 

As for granodiorite, it is an igneous rock rich in silica and alumina oxides, which impart pozzolanic activity when finely ground [56]. At the nanoscale, NG exhibits a highly reactive surface and a filler effect that enhances the formation of secondary C–S–H gel and improves particle packing within the cement matrix. These characteristics contribute to a denser and more thermally stable microstructure, capable of resisting the adverse effects of high-temperature exposure. NG is also used for the first time in the present study with the objective of improving the post-heating compressive strength and thermal durability of concrete, where it showed promising results when used as a micro-size partial replacement material of cement in earlier study [57]. It is worth mentioning that the present study represents an extension of a previous experimental research program in which the current nanomaterials were investigated for their effects on several properties of concrete under normal conditions [58]. The findings reported in the literature study indicated that NG exhibited superior enhancement of the mechanical properties of concrete due to its pronounced pozzolanic activity combined with a strong filler effect.

RSM integrates mathematical and statistical techniques to establish relationships between multiple independent variables and the measured response, thereby facilitating the identification of significant factors, parameter interactions, and optimal conditions. The user-defined approach [59] allows for model development utilizing an experimental matrix. Numerous studies have successfully utilized RSM to optimize concrete formulations, develop predictive models, and reduce experimental complexity [60,61,62,63,64,65], highlighting its effectiveness as a robust optimization and modeling tool in material science research. To comprehensively evaluate and optimize the influence of the used nanoparticles on the performance of concrete under high temperatures, a combined experimental and statistical modeling approach based on the response surface methodology (RSM) was employed to analyze the effects of temperature, nanomaterial type, and nanoparticle dose on the residual compressive strength of concrete subjected to high temperatures. RSM’s optimal custom design (OCD) technique in RSM was utilized for CS simulation. To examine the models’ performance, analysis of variance (ANOVA) was utilized. Additionally, numerical optimization was used to optimize nano-modified concrete properties at high temperatures. 

Despite considerable progress in the use of nanomaterials to improve the high-temperature performance of concrete, the available literature remains limited regarding the effects of NL, particularly in terms of residual compressive strength following elevated-temperature exposure. Although previous studies have demonstrated the effectiveness of NL in enhancing the gamma-ray attenuation capacity of concrete and investigated its influence on concrete properties under conventional conditions, its potential contribution to preserving mechanical performance after heating remains insufficiently explored. Similarly, the high-temperature performance of NG, which has shown promising mechanical benefits under normal conditions, has not been adequately established. Although NM has been investigated in cement-based materials, further comparative evidence is needed to clarify its effectiveness relative to other nanomaterials under thermal exposure. Furthermore, the combined influence of nanomaterial type, dosage, and exposure temperature on residual compressive strength requires systematic evaluation to identify effective mixture proportions and establish reliable predictive relationships. To address these research gaps, the present study experimentally investigates the residual compressive strength of concrete incorporating NL, NM, and NG as partial cement replacement materials following exposure to elevated temperatures. The effects of nanomaterial type, replacement dosage, and heating temperature are evaluated using RSM. The novelty of this study lies in the comparative assessment of these three nanomaterials within a unified experimental and statistical framework, with particular emphasis on investigating the largely unexplored potential of NL and the first reported application of NG at the nanoscale for improving the residual compressive strength of concrete after heating. The resulting models are used to elucidate the relationships between the investigated variables and identify mixture conditions that maximize residual compressive strength.

## 2. Materials Properties

Building upon prior work, this study extends and expands our previous experimental program, which investigated the effects of nano lead oxide (NL) and nano granodiorite (NG) on the mechanical strength of high-strength concrete [58]. The current research broadens the material scope by incorporating NM. The present study also extends its investigation to the influence of elevated temperatures on the behavior of concrete incorporating the three aforementioned nanomaterials. This allows for the evaluation of mix performance under severe environmental conditions. The effects of this specific incorporation under elevated temperatures are being explored for the first time in the current research. The motivation for including NM with the nanomaterials investigated in this study stems from its intermediate properties relative to lead oxide and granodiorite. While lead oxide is a high-density material that serves as an inert filler, granodiorite in both bulk and nanoparticle size offers a relatively lower density and exhibits pozzolanic activity, thereby enhancing the pozzolanic reactions within the cementitious system. Magnetite has a moderate density between these two materials and, and its application in concrete mixes in the nanoscale of particles can combine dual beneficial mechanical properties. However, its influence on the mechanical behavior of concrete, especially under elevated temperature exposure, has been rarely explored in previous investigations. This section presents the properties of the constituent materials used in the concrete mixtures, including cement, aggregates, water, and superplasticizer, as well as the characteristics of the nanomaterials employed in the study. The latter include nano-silica (NS), used as a constant additive in all concrete mixtures. The other nanomaterials of NL, NM, and NG, were incorporated as partial replacements of cement by weight.

### 2.1. Cementitious Materials

**Figure 1:**
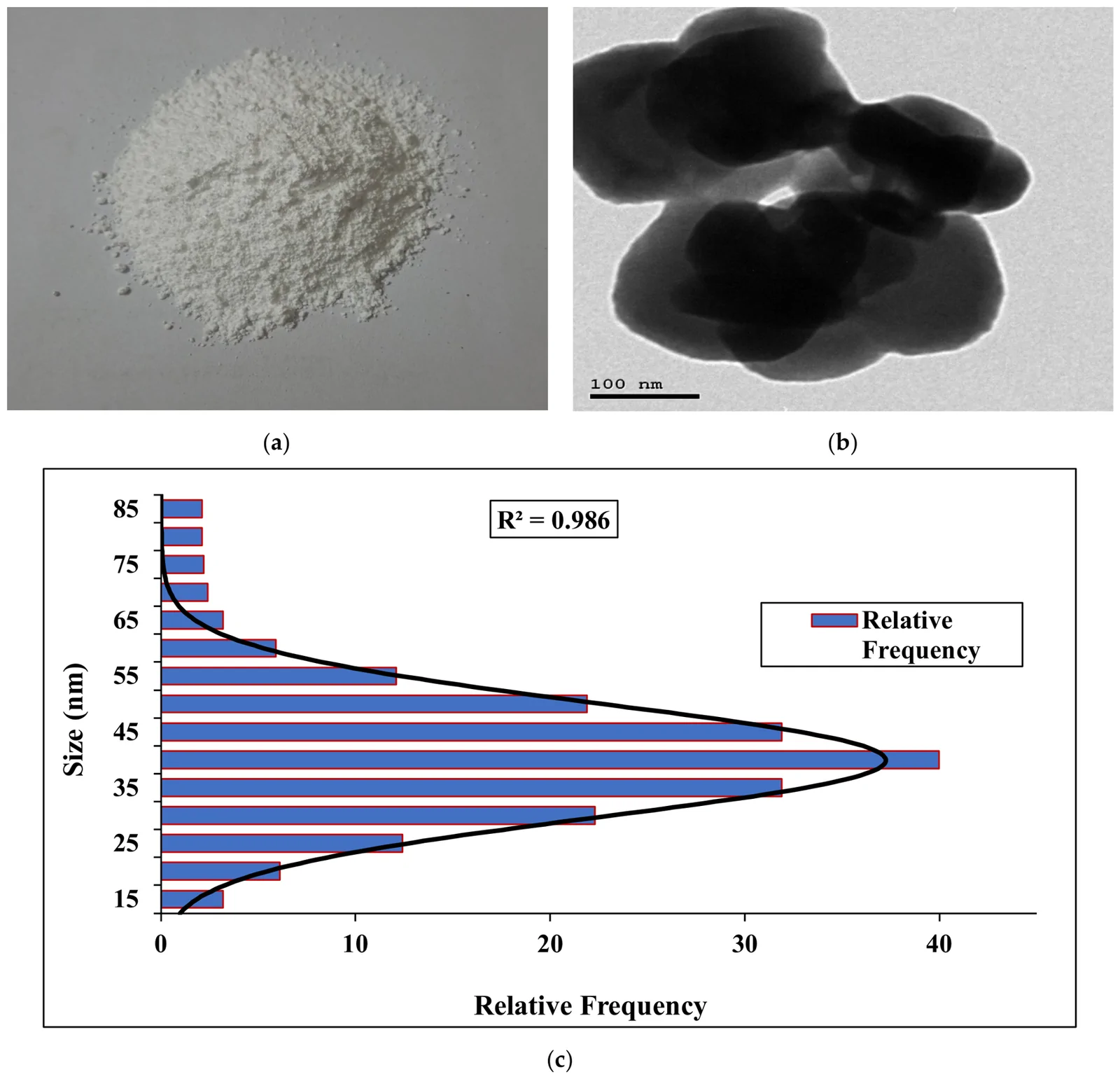
Properties of NS (**a**) appearance, (**b**) TEM image, and (**c**) particle size distribution.

Ordinary Portland cement (CEM I 52.5 N) was used as the main binder, conforming to the requirements of the European Standard EN 197-1 [66] for Portland cement, with a dosage of 500 kg/m^3^ in the control mix. In addition, NS was incorporated as a supplementary cementitious material at a constant replacement level of 2% by cement weight in all mixtures, including the control mix. The cement has a relative density of 3.15 and initial and final setting times of 150 and 235 min, respectively. Standard mortar cube testing per EN 196-1 [67] yielded compressive strengths of 27.3, 37.5, and 53.4 MPa at 3, 7, and 28 days, respectively. A gel-type nano-silica that replaced 2% of the cement by weight was used in all mixtures. Table 1 presents the chemical composition of the cement used in the present study, in addition to the nanomaterials incorporated. Figure 1 delineates the comprehensive physicochemical characterization of the NS employed throughout the present investigation. The characterization procedure encompasses multiple analytical techniques, including visual documentation of the sample appearance, transmission electron microscopy (TEM) for nanoscale morphological evaluation, analysis for crystallographic phase determination, and systematic particle size distribution assessment.

### 2.2. Aggregates

This experimental program used dolomite-based coarse aggregate and fine silica sand carefully selected to meet parameters. The coarse aggregate had a bulk density of 1660 kg/m^3^, specific gravity of 2.65, a maximum nominal size of 19 mm, water absorption capacity of 0.45%, and a crushing value of 22%. The fine aggregate had a bulk density of 1730 kg/m^3^, a specific gravity of 2.55, a water absorption of 0.9%, and a fineness modulus of 2.46. The particle size distribution showed that both coarse and fine aggregate fractions consistently met EN 12620 [68] upper and lower gradation boundaries, proving their suitability for producing high-quality concrete mixtures.

### 2.3. Water and Superplasticizer (Sp)

Clean potable water complying was utilized for mixing and curing operations. The water-to-binder materials ratio was 0.32. Locally available polycarboxylate-based superplasticizer conforming to EN 934-2 [69] specifications was incorporated at a dosage of 1.8% by weight of cement.

### 2.4. Nano Materials

#### 2.4.1. NL Properties

**Figure 2:**
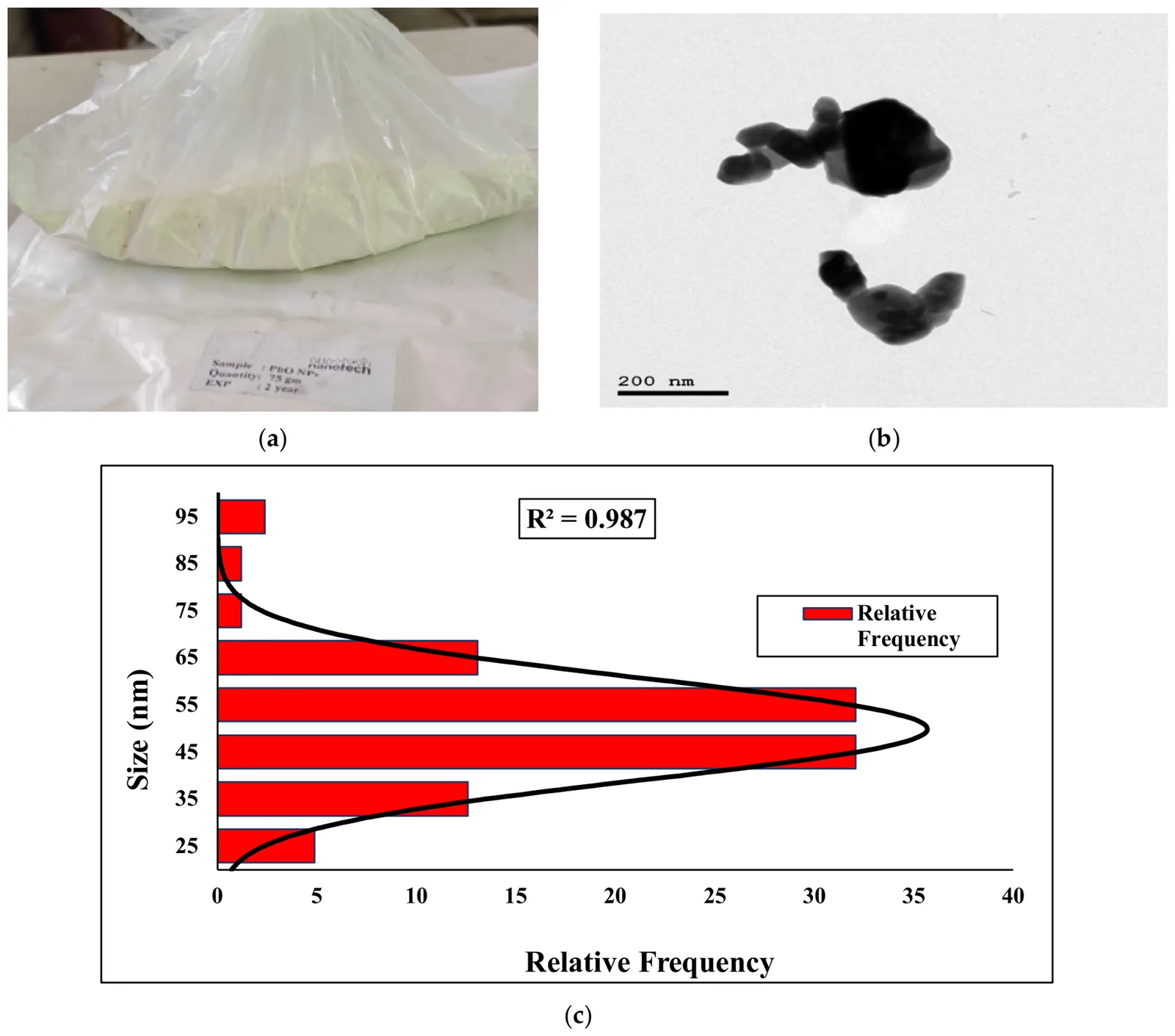

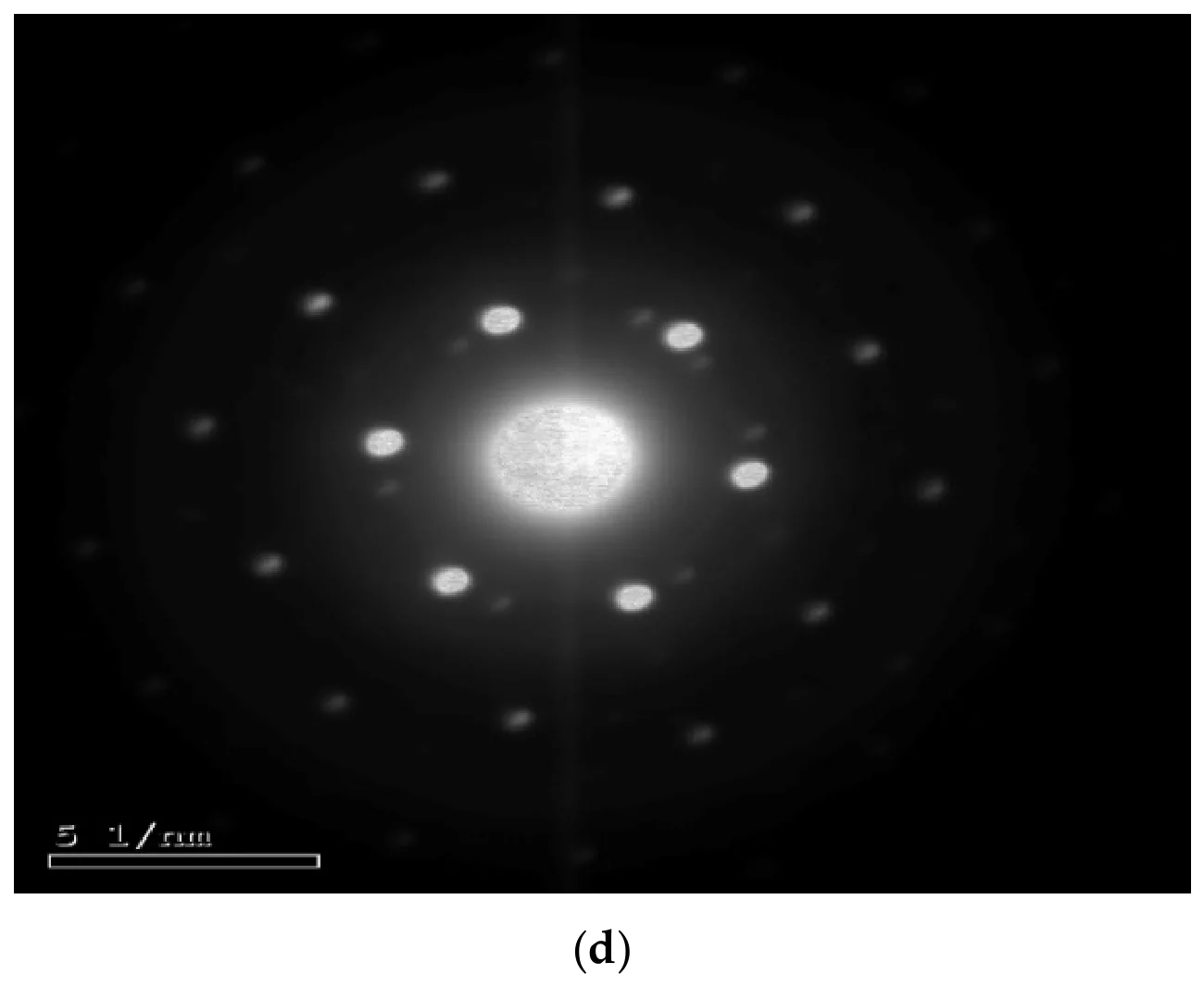
Properties of NL (**a**) appearance, (**b**) TEM image, (**c**) particle size distribution, and (**d**) SAED pattern.

The lead oxide (PbO) nanoparticles employed in this investigation were synthesized by Nanotech company, a leading local nanomaterial manufacturer in Egypt. Figure 2a displays the macroscopic appearance of the nanoparticles, exhibiting a characteristic bright white coloration. Figure 2b presents the TEM micrograph revealing irregularly shaped, electron-dense PbO nanoparticles at the nanoscale. Particle size distribution analysis (Figure 2c) determined an average particle size of 50.38 nm, with the majority of particles concentrated in the 45–55 nm range. The selected area electron diffraction (SAED) pattern in Figure 2d shows concentric rings of discrete diffraction spots, confirming the polycrystalline nature and high crystallinity of the nanoparticles.

#### 2.4.2. NM Properties

**Figure 3:**
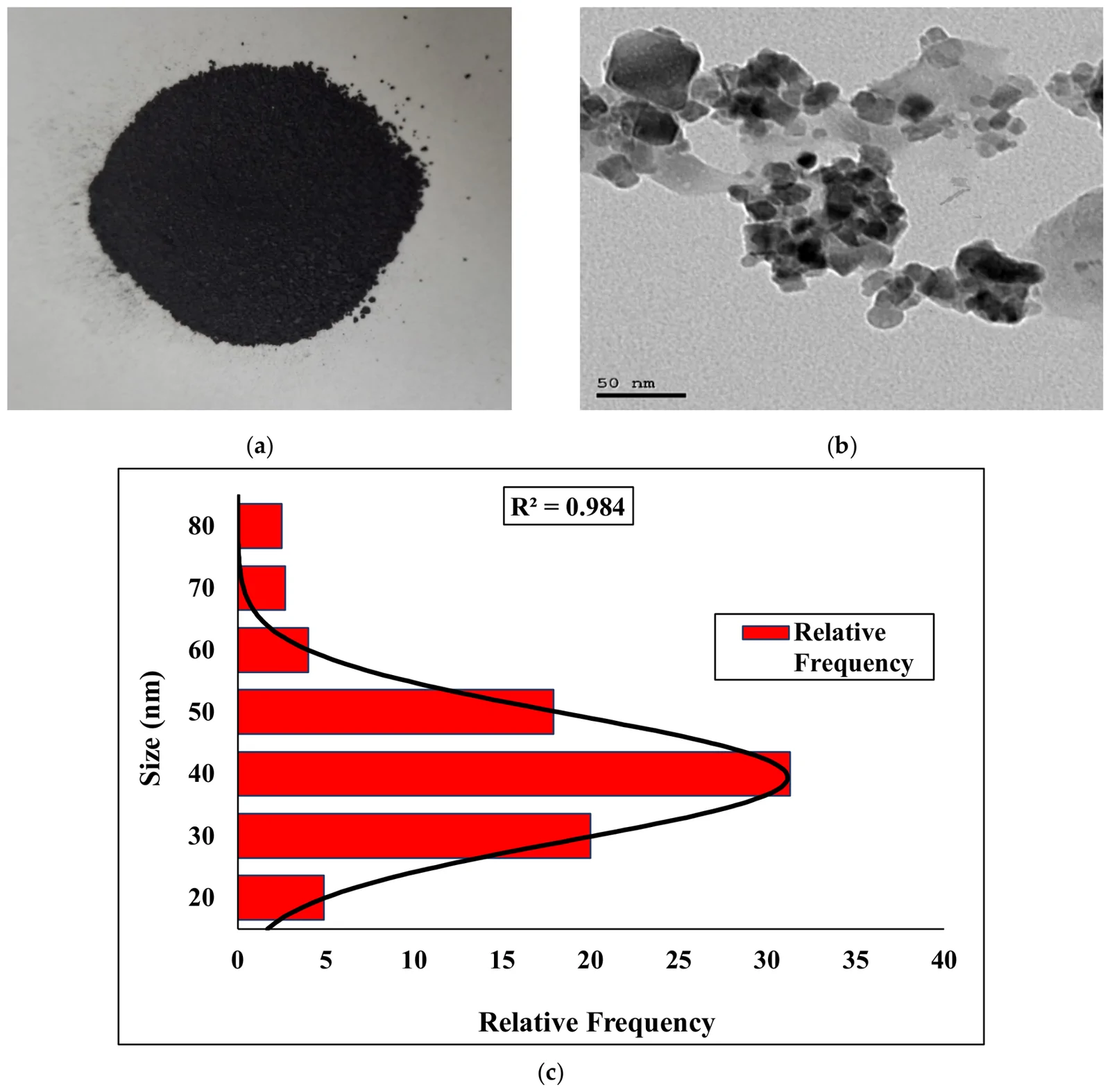

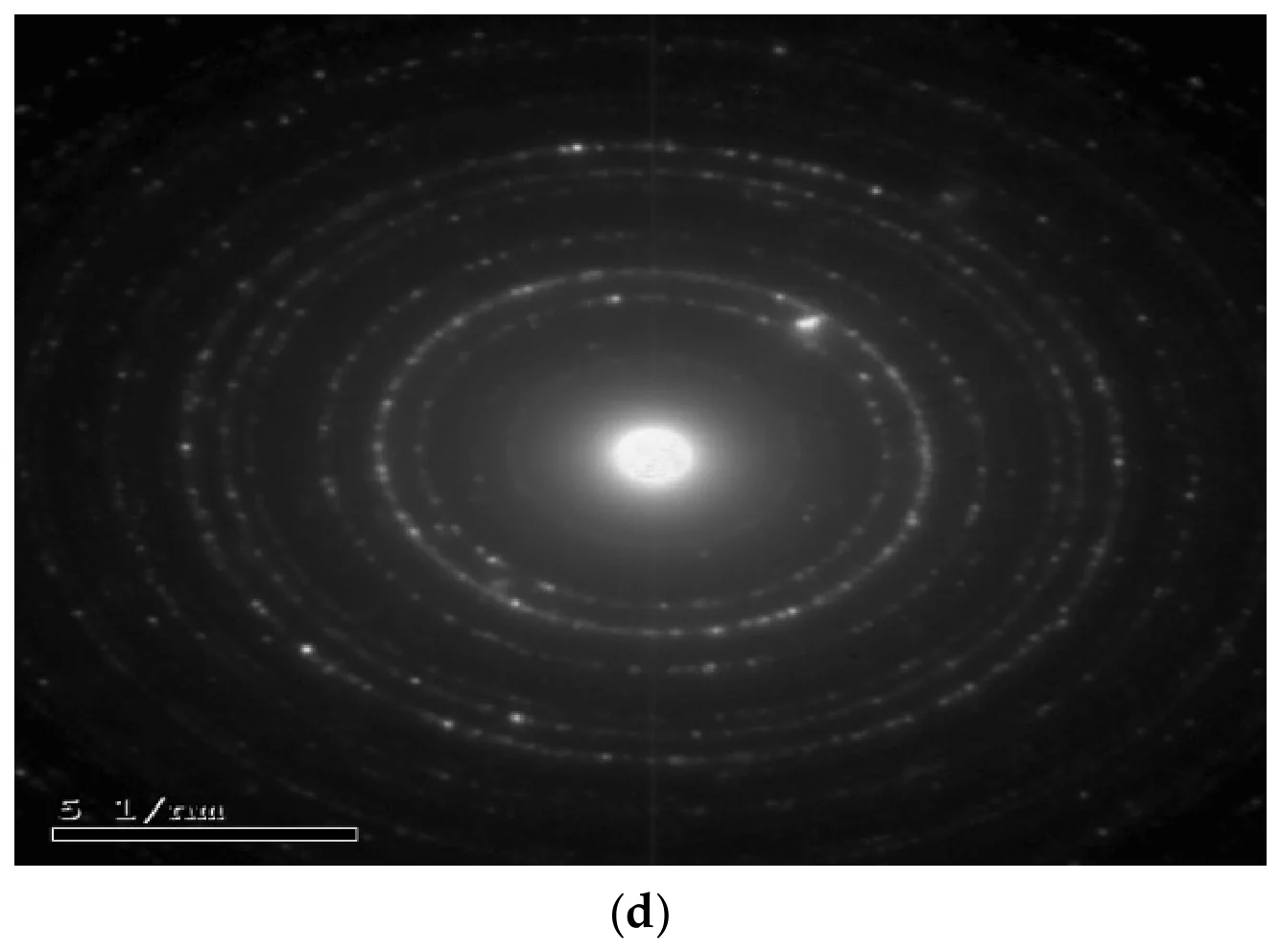
Properties of NM (**a**) appearance, (**b**) TEM image, (**c**) particle size distribution, and (**d**) SAED pattern.

Nano-magnetite (Fe_3_O_4_) is a high-purity NM particle with a density of 5.17 g/cm^3^. Figure 3 presents the comprehensive characterization of the nano-magnetite employed in this investigation. Figure 3a displays the appearance of the NM powder, showing its characteristic black coloration typical of magnetite nanoparticles. Figure 3b presents the TEM micrograph revealing irregularly shaped, highly electron-dense nanoparticles with a tendency toward agglomeration, as evidenced by the clustered arrangement of particles. The 50 nm scale bar confirms the nanoscale dimensions of individual particles. Figure 3c presents the histogram of particle size distribution, revealing an average particle size of 44.5 nm, with most particles concentrated in the 40–50 nm range, demonstrating a relatively narrow size distribution suitable for uniform dispersion in cementitious matrices. Figure 3d shows the SAED pattern, characterized by well-defined concentric diffraction rings, confirming the polycrystalline nature and crystalline structure of the magnetite nanoparticles. The sharp, continuous rings indicate random orientation of multiple crystalline domains within the sample.

#### 2.4.3. NG Properties

**Figure 4:**
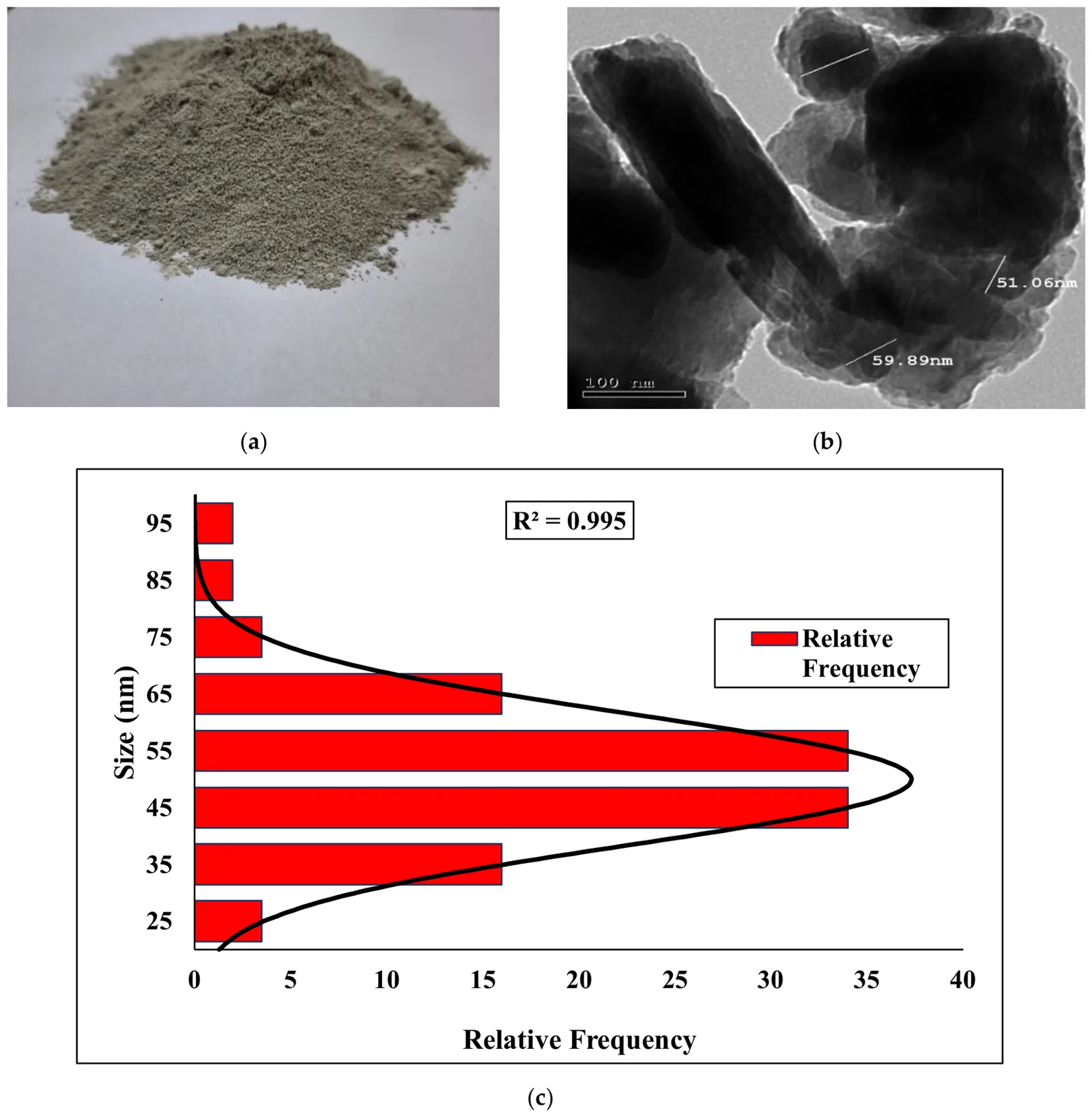

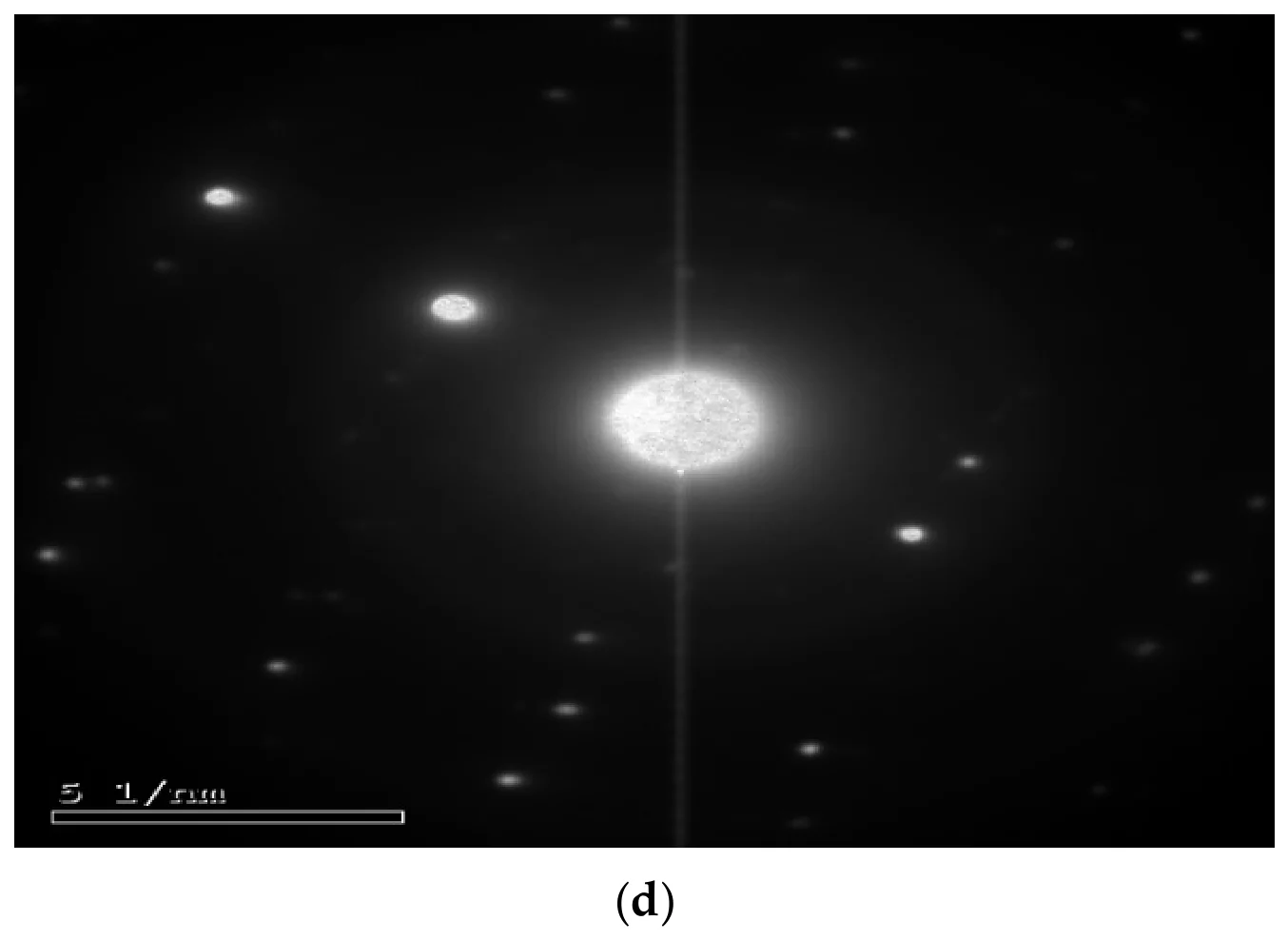
Properties of NG (**a**) appearance, (**b**) TEM image, (**c**) particle size distribution, and (**d**) SAED pattern.

Granodiorite rock samples were reduced to nanoscale size by planetary ball milling. Following the first crushing, the material was ground with hardened steel balls at 370 RPM for 17 h under optimal circumstances. The characterization of the resulting NG is presented in Figure 4. Figure 4a shows the macroscopic appearance of the fine, gray NG powder. TEM analysis (Figure 4b) reveals irregularly shaped, angular particles at the sub-micron to nanoscale level, characteristic of mechanically milled crystalline materials. Particle size distribution analysis (Figure 4c) demonstrates that the NG powder achieved an average particle size of 52.7 nm, with the predominant fraction concentrated in the 45–55 nm range. The SAED pattern (Figure 4d) displays discrete diffraction spots, confirming that the NG particles maintain their crystalline structure post-milling.

## 3. Experimental Program

### 3.1. Mixes Design

The control mix proportions, as delineated in Table 2, show the concrete ingredients. An experimental program encompassing fifteen modified mixtures was developed to systematically evaluate the effects of nanomaterials as partial cement replacements at varying substitution rates. Nano-silica (NS) was incorporated at a constant replacement level of 2% by cement weight in all mixtures, including the control mix. The experimental program included three distinct series of mixtures: five mixtures incorporating nano-PbO (NL) as an additional partial cement replacement at dosages of 1%, 2%, 3%, 4%, and 5% by weight of cement; five mixtures employing nano-Fe_3_O_4_ (NM) at the same replacement percentages; and five mixtures utilizing nano-granodiorite (NG) at identical substitution levels. The quantities of coarse and fine aggregates were 1130 kg/m^3^ and 695 kg/m^3^, respectively. A constant water-to-cementitious-material ratio of 0.32 was maintained, while the superplasticizer dosage was 9 kg/m^3^.

### 3.2. Specimens Preparation and Curing

Concrete specimens were prepared using the mixture design proportions specified in Table 2 to measure compressive and splitting tensile strength parameters. Steel molds were used to cast cylindrical specimens of 100 mm in diameter and 200 mm in height, as well as cubic specimens measuring 100 mm × 100 mm × 100 mm. A wet mixing methodology was used to achieve uniform distribution of all elements, particularly nanoparticles (NS, NL, NM, and NG), which is crucial for preventing ultrafine particle agglomeration inside the cementitious matrix. The mixing procedure began with the addition of dry ingredients such as cement, coarse aggregate, and fine aggregate to the mixer in the specified amounts, followed by 3 min of uniform dry blending. The examined nanomaterial (NL, NM, or NG) was then disseminated in 80% of the total design water content and subjected to high-speed magnetic stirring for 2 min to ensure optimal particle dispersion. The nanomaterial-water suspension was then progressively added to the main mixer. The remaining 20% of the water was mixed with the NS additive and the necessary SP dosage, violently stirred for 2 min with the magnetic stirrer, and then gradually added to the mixer. All concrete constituents were then mixed for around 3 min to guarantee thorough homogeneity of the product. After mixing, the fresh concrete was cast into the prepared molds and subjected to mechanical vibration to eliminate entrapped air gaps, maintain proper compaction, and produce a consistent surface quality. The specimens were then coated with polyethylene sheets to prevent moisture evaporation and kept under ambient laboratory conditions for 24 h before demolding. After demolding, all specimens were fully submerged in a temperature-controlled water bath maintained at 23 ± 2 °C with 95% relative humidity for 28 days. Upon completion of the 28-day hydration period, specimens designated for elevated temperature exposure were extracted from the curing tank and allowed to air-dry at ambient laboratory conditions until achieving a surface-dry state, thereby ensuring consistent initial moisture conditions prior to subsequent thermal exposure and mechanical characterization.

### 3.3. Elevated Temperatures Exposure

Concrete cubic samples were exposed to a thermal effect procedure at three temperature levels: 400 °C, 600 °C, and 800 °C. Each temperature plateau was maintained for a constant duration of 120 min to ensure uniform thermal penetration throughout the specimens. The thermal conditioning was conducted using a custom-designed electric furnace. The furnace assembly was fabricated from welded steel plates reinforced with steel angles at the periphery, incorporating a 7 cm cavity filled with glass wool insulation to minimize thermal losses. The internal chamber, with dimensions of 45 cm × 45 cm × 120 cm, was equipped with eight electrical heating elements capable of achieving a maximum operating temperature of 1200 °C. Temperature regulation and monitoring were achieved through an automated control system integrated with a thermocouple sensor and digital display interface, which continuously recorded the actual internal chamber temperature in real-time. The furnace’s thermal performance was validated against the ISO 834 standard fire resistance curve, which can be defined by Equation (1) [70,71], to ensure compliance with internationally recognized testing protocols.

Temperature degree = 20 + 345.log(8t + 1)(1)

where t is the time in minutes.

**Figure 5:**
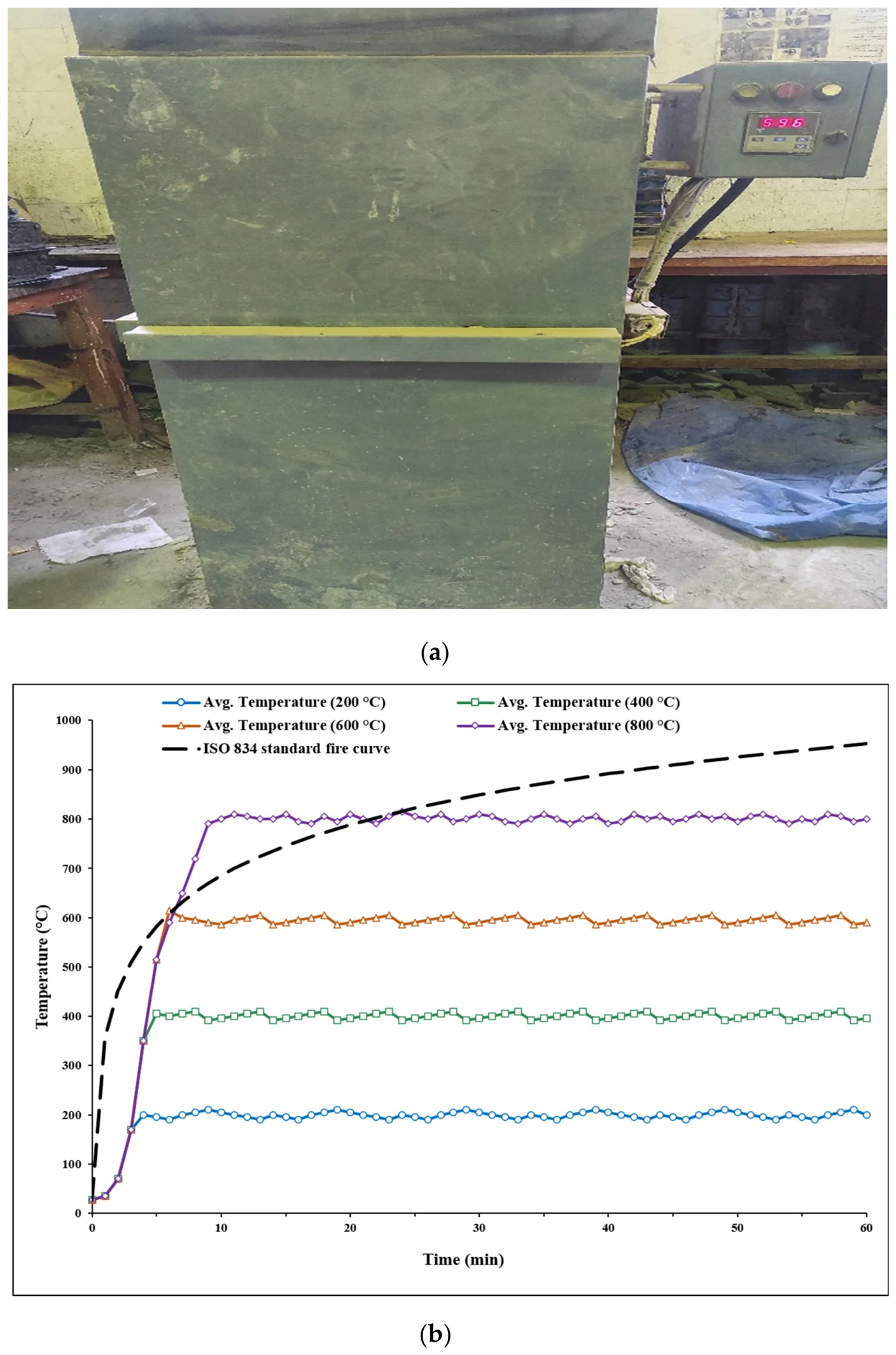
(**a**) The electric muffle furnace used for thermal exposure and (**b**) the measured furnace temperature profile compared with the ISO 834 standard fire curve.

The experimental setup utilized an electrical muffle furnace, shown in Figure 5a, equipped with a digital temperature screen for precise control and monitoring of the thermal regime. The furnace was controlled to maintain the target temperatures for the constant exposure time of 120 min. The resulting time—temperature curve generated by the test furnace, particularly the 800 °C average temperature profile, demonstrates a close and effective correspondence to the mandated ISO 834 curve, as shown in Figure 5b. The measured furnace temperature initially exhibits an aggressive heating rate, briefly surpassing the ISO 834 standard around the 8 to 10-min mark. This rapid initial attainment of high temperature validates the furnace’s high thermal responsiveness and control system fidelity. Subsequently, the measured 800 °C curve effectively tracks the standard, confirming that the controlled thermal environment replicates the required severe fire exposure necessary for accurate fire resistance classification.

### 3.4. Compressive Strength Test

The comprehensive mechanical testing was conducted using 3000 kN capacity ELE ADR digital compression testing machine, equipped with a microprocessor-controlled readout. The mechanical behavior of the concrete mixtures was measured after a 28-day curing period to reassess the compressive strength of specimens exposed to elevated temperatures following air cooling to ambient conditions. Two principal tests were carried out: the ultimate compressive strength, determined using 100 × 100 × 100 mm cubic specimens, and the splitting tensile strength, obtained by applying a continuous compressive load along the horizontal axis of cylindrical samples until failure. The digital interface of the testing unit provides real-time load monitoring (in kN, lbf, or kgf), ensuring operator control over the standardized loading rate, while automatically calculating stress values based on the input specimen dimensions. Furthermore, the system improves data accuracy and traceability by recording each test result together with the machine’s serial number and load–time cycle plot, thereby ensuring a consistent and verifiable evaluation of the mechanical properties of all tested concrete formulations, both before and after thermal exposure. The compressive strength was calculated using the following Equation:

CS = P/A(2)

where

P = the maximum applied load at failure (N);
A = the cross-sectional surface area of the cube specimen (mm^2^).

## 4. Experimental Results

### 4.1. Compressive Strength at Ambient Temperature

**Figure 6:**
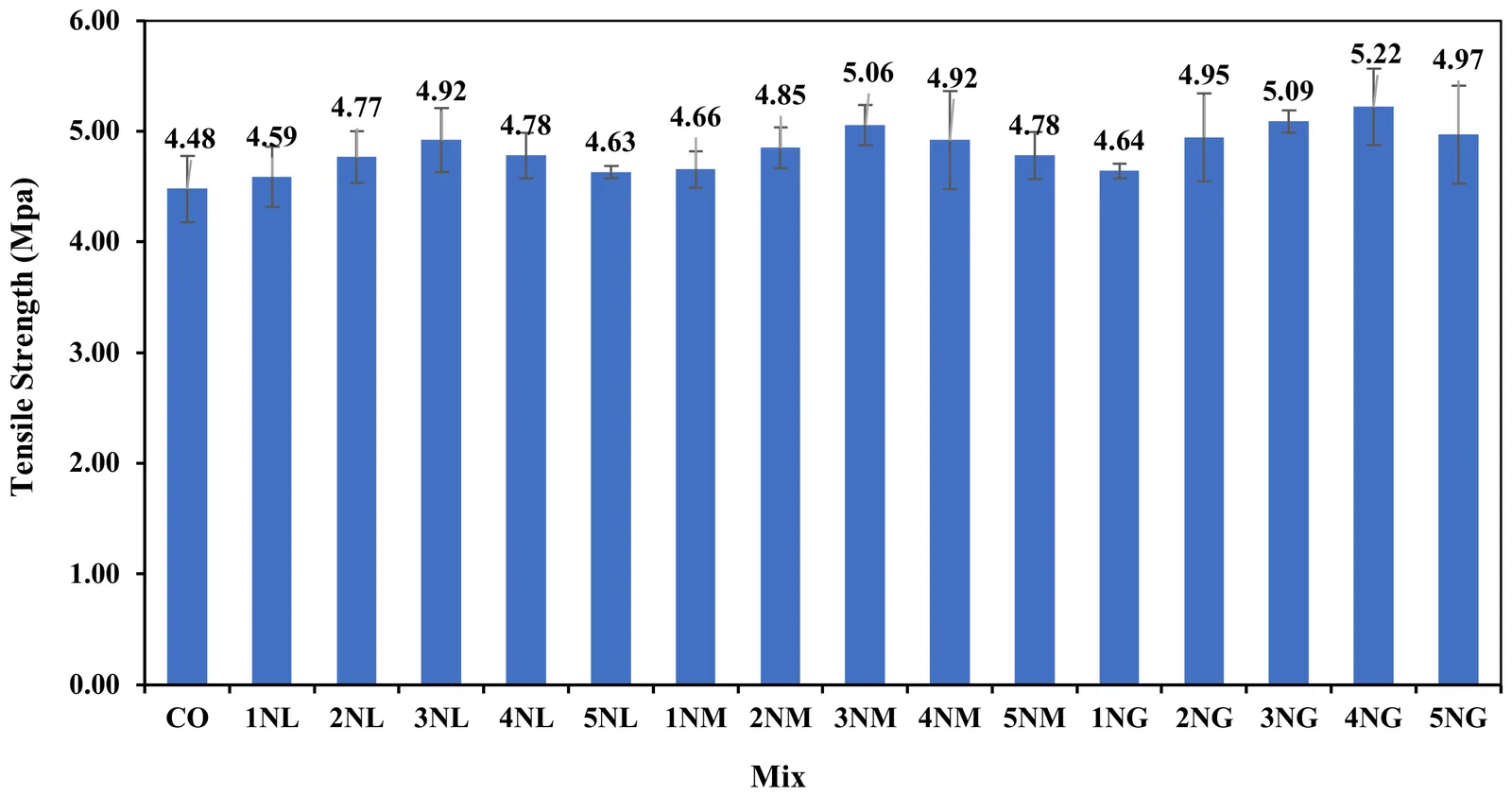
Tensile strength for the investigated mixes at traditional conditions.

As previously noted, the current study serves as an extension of a prior investigation in which the effects of replacing cement with 1–5% by weight of NG, NM, and NL were examined on several properties of concrete under normal conditions [58]. The findings reported in the literature demonstrated an enhancement in both the compressive and tensile strengths of concrete containing these nanomaterials at various replacement levels. The optimal replacement ratios were found to be 4% for NG and 3% for NL, at which compressive strength improved by approximately 17.3% and 8%, respectively, while tensile strength increased by 16.5% and 9.8%, respectively. These improvements can be attributed to different mechanisms. NG acts as a pozzolanic material due to its high content of silicon and aluminum oxides, which exhibit higher reactivity at the nanoscale, thereby contributing to the formation of additional calcium silicate hydrate (C–S–H) gel that enhances the microstructural compactness and mechanical strength of the concrete matrix. Furthermore, the inert particles of NG may contribute to pore refinement by filling micro voids within the concrete matrix due to their ultra-fine particle size and high surface area, which improve particle packing density and reduce internal porosity. Regarding NL, although it does not directly participate in pozzolanic reactions, its fine particle size enhances the filler effect, leading to a denser microstructure and improved interfacial transition zone (ITZ) between the cement paste and aggregates. This densification mechanism contributes to better mechanical integrity and reduces microcracking within the hardened concrete. Regarding NM, its performance was moderate compared with that of NL and NG. The optimal replacement level for compressive strength was observed at 4%, resulting in an enhancement of 11.2% compared to the control mix. In the case of tensile strength, the optimum replacement ratio was 3%, which led to an enhancement of about 12.8% relative to the control mix. The improvement in strength observed in NM-modified concrete can be attributed to several mechanisms. Nano magnetite particles possess a high surface area and strong interfacial bonding capability, which enhances the adhesion between the cement paste and aggregate particles, leading to a denser and more homogeneous microstructure. In addition, NM can act as a nucleation site that accelerates the hydration process of cement, promoting the formation of additional C–S–H gel and improving microstructural compactness. Its high density and magnetic nature may also contribute to refining the pore structure, minimizing microcrack propagation, and improving the load transfer efficiency within the matrix. Mechanical properties’ values of both compressive and tensile strength at ambient temperature condition for all investigations concrete mixes are introduced in Table 3 and Figure 6.

### 4.2. Effect of Elevated Temperature on Concrete Compressive Strength

**Figure 7:**
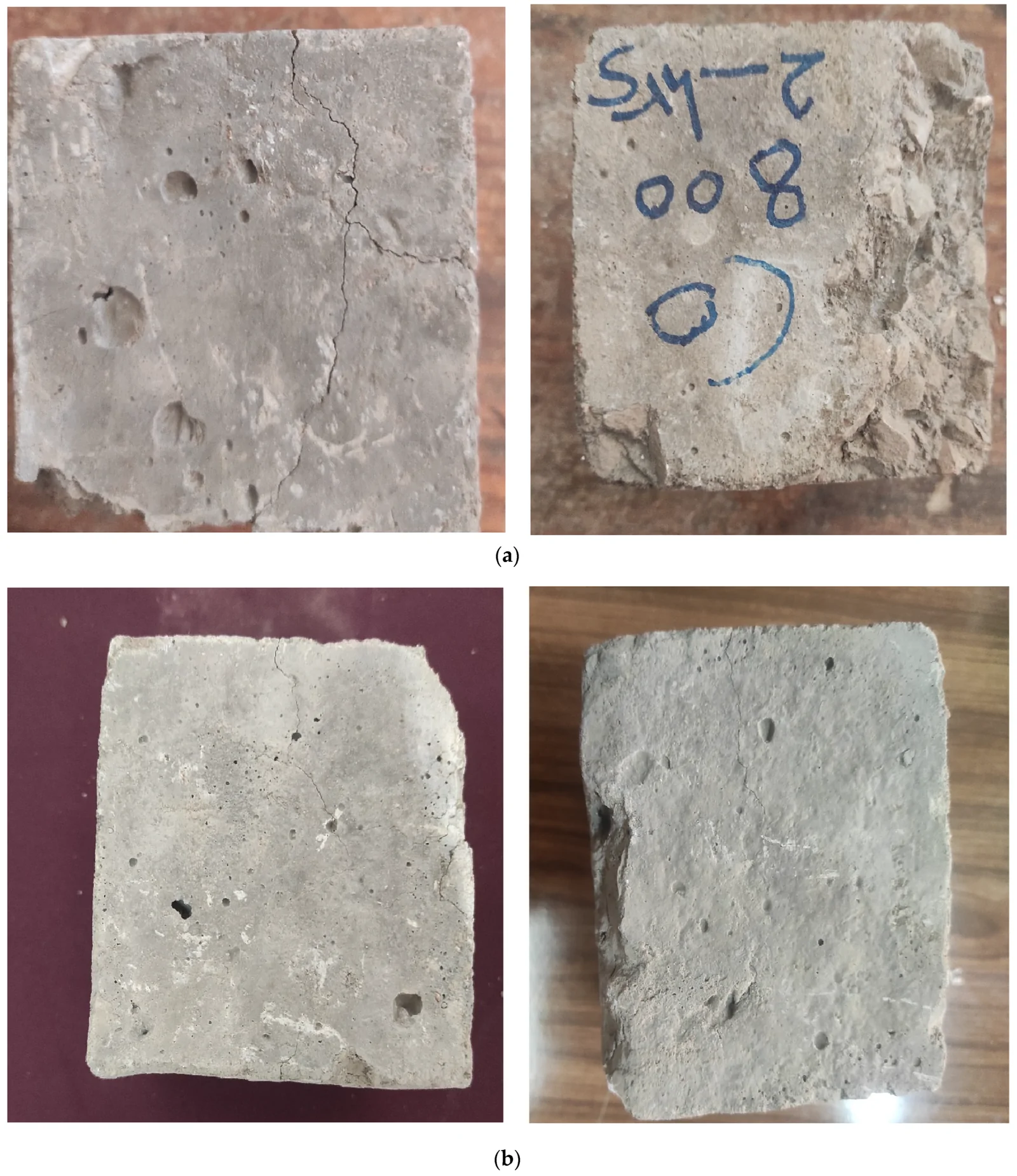
Concrete specimens after exposure to 800 °C: (**a**) control mix and (**b**) 4%NG mix.

Results of the residual compressive strength values after elevated temperature exposure are presented in Table 3. Regarding exposure to high temperatures, the concrete cubes were subjected to a constant heat exposure time of 120 min in electric furnace, with varying temperatures of 400 °C, 600 °C, and 800 °C. The results showed that the tested nanomaterials were capable of improving the concrete’s resistance to the effects of extreme conditions of high temperatures exposure with varying degrees of improvement. Exposure to high temperatures resulted in a significant reduction in the compressive strength of concrete. This effect was particularly pronounced at 800 °C, where the control mix exhibited a strength loss exceeding 48.39% compared to its room temperature value. Figure 7a illustrates the effect of exposure to 800 °C on the control mix concrete cube, showing clear signs of thermal deterioration. The surface appears rough and heavily spalled, particularly along the edges, indicating the breakdown of the cement paste and aggregate–paste bond under thermal stress. The surface texture further suggests extensive microcracking and void formation caused by differential thermal expansion and dehydration of cementitious components. Figure 7b illustrates the optimal concrete mix containing 4% NG replacement after exposure to 800 °C. The NG-modified cubes retain a smoother, more intact surface with only minor edge deterioration and fewer, finer cracks. The surface of the NG mix also appears denser and less porous, suggesting that the nano-filling and pozzolanic effects of NG contributed to a more compact microstructure capable of withstanding thermal stress. Despite the significant improvement in strength compared to the control mix, the effect of high temperature remains evident, as the optimal 4% NG mix still lost approximately 39.23% of its original compressive strength after exposure to 800 °C for two hours. Several factors contribute to the decreased concrete strength under these conditions. These factors include water evaporation, degradation of hydration compounds and aggregates, microstructure expansion, mass loss, and increased porosity [11,72]. Furthermore, the possibility of spalling due to increased water vapor pressure and thermal stresses exacerbates the degradation of the concrete’s mechanical properties, particularly its compressive strength, when exposed to high temperatures [73].

**Figure 8:**
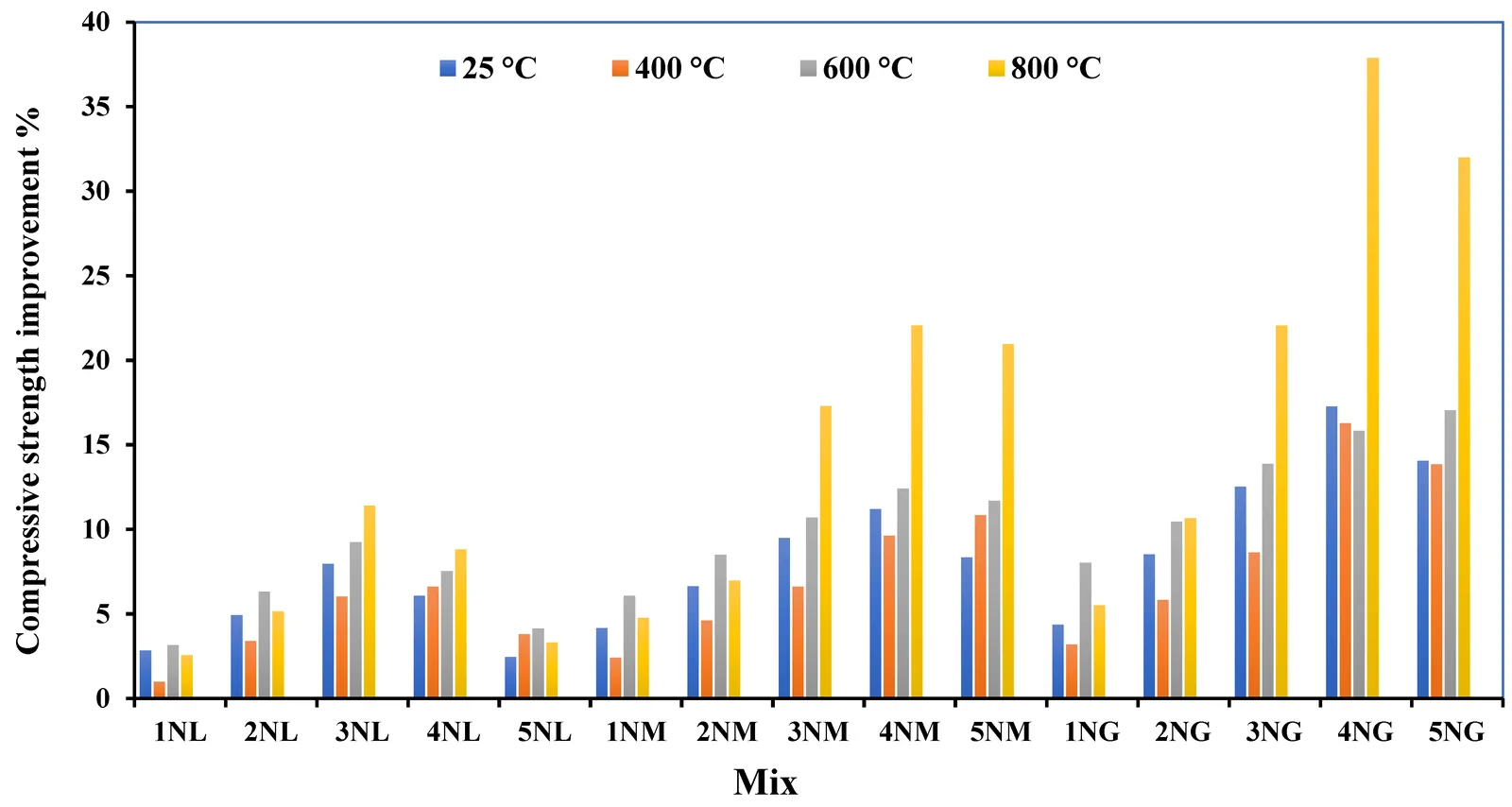
Residual compressive strength improvement rates regarding the control mix for all concrete samples investigated.

Nanomaterials offer a promising avenue for enhancing the performance of concrete subjected to elevated temperature [74]. By acting as nucleation sites for hydration products and improving interfacial bonding, nanoparticles can create denser and more compact microstructures that are better able to resist thermal degradation [30]. Additionally, nanomaterials can reduce porosity through the nano filling effect, minimizing the penetration of harmful agents that can accelerate deterioration at elevated temperatures [75]. Results of compressive strength improvement rates compared to the control mix after extreme conditions exposure are cleared in Figure 8.

Among the nanomaterials investigated as cement substitutes, NG demonstrated the most promising results in enhancing the residual compressive strength of concrete exposed to high temperatures. An optimal replacement ratio of 4% yielded a substantial 37.87% improvement in compressive strength compared to the control mix when subjected to 800 °C. NM exhibited the second-highest efficacy in mitigating the detrimental effects of high temperature, with a 4% replacement ratio leading to a 22.06% increase in residual compressive strength. While NL displayed the least effectiveness in improving the concrete’s resistance for high-temperature exposure effects, it nevertheless provided a modest enhancement, particularly at a 3% replacement level, where an 11.4% increase in compressive strength was observed at 800 °C. The superiority of using NG particles compared to the other two nanomaterials lies in its dual effect, which contributes to enhancing concrete’s resistance to the impacts of high temperatures exposure. In addition to the well-known void filling effect of most nano additives, the pozzolanic activity of NG powder aids in activating hydration reactions, leading to an increase in strength as well as a reduction in micro-cracking.

## 5. Surface Response Method Model

### 5.1. Introduction to Response Surface Methodology (RSM) Method

Design-Expert software (Version 13) was employed to implement the Optimal Custom Design (OCD). Among the various RSM design methodologies, OCD is the most widely used and straightforward for modelling and optimization in concrete research [76]. In this design, the independent variables are correlated with the response through linear, interaction, or quadratic polynomial functions. Equation (3) represents the general form of the second-order polynomial used in RSM to describe the relationship between the independent variables and the response. In the present study, ANOVA was applied to the experimental data to assess the statistical significance of each factor and identify the parameters that exert the greatest influence on the compressive strength (CS).

(3) $$\mathrm{Predicted}\;\mathrm{response}=\omega_{o}+\sum_{i=1}^{m}\omega_{i}X_{i}+\sum_{i=1}^{m}\omega_{\mathrm{ii}}X_{i}^{2}+\sum \sum_{i<j}{\omega_{\mathrm{ij}}X}_{i}X_{j}$$

where *ω_o_* is the intercept, *ω_i_* is the linear coefficients, *ω_ii_* is the quadratic coefficients, *ω_ij_* is the interaction coefficients, and *X_i_*, *X_j_* are the independent factors.

### 5.2. Analysis of Variance (Anova)

The use of ANOVA within the RSM framework was critical in measuring the individual and interaction impacts of input parameters—nanomaterial type, replacement rate, and temperature degree—on concrete compressive strength values. The generated regression model was statistically evaluated to ensure its significance and dependability. The model’s high F-value and associated *p*-value of less than 0.05 confirmed that the model was statistically significant and the terms were not caused by random noise. The goodness of fit was tested using the coefficient of determination. The adjusted R^2^ value, which accounts for the number of predictors, was close to the anticipated R^2^ value, indicating a robust model with a low risk of overfitting. A high enough precision value, as measured by the signal-to-noise ratio, demonstrated the model’s ability to navigate the design space. Furthermore, all analyses were performed at a 95% confidence level, which meant that the observed relationships had a 5% chance of occurring by chance. Collectively, these statistical indicators confirmed the model’s excellent predictive accuracy for refining nanomaterial concrete formulations for improved elevated temperature resistance.

The ANOVA results in Table 4 indisputably demonstrate the high statistical significance and robust predictive capability of the constructed regression models for the compressive strength of nano-modified concrete. For all three nanomaterials, the extremely high F-values and corresponding *p*-values of less than 0.0001 firmly reject the null hypothesis, confirming that the modelled terms (nanomaterial dose and temperature) have a statistically significant effect on the compressive strength response and that the models are highly significant, with a near-zero probability that this signal is due to noise. With R-values exceeding 0.99, the models accurately fit the experimental data, accounting for over 99% of the variability in compressive strength. The strong agreement between adjusted and predicted R^2^ values for each model demonstrates that they are not overfit and have excellent predictive potential for new data. This is further confirmed by the high Adequate Precision values, all of which are considerably over the acceptable threshold of 4, indicating a high signal-to-noise ratio and confirming the models’ fitness for navigating the design space. The low C.V.% results, especially for nano-lead (1.39%) and nano-magnetite (1.45%), indicate great reliability and minimum experimental error in comparison to the mean response. Collectively, these statistical indicators provide a high level of confidence in the models’ suitability for optimization and industrial application.

### 5.3. Prediction of Concrete Compressive Strength

#### 5.3.1. Regression Models

The constructed second-order polynomial regression models, as stated in Equations (4)–(6), quantitatively explain the compressive strength (CS) of nano-modified concrete as a function of nano-dose (N) and temperature. All three equations capture the complexity and nonlinearity of the strength response. The positive linear coefficients for N in all equations demonstrate that, within the studied range, increasing the nano-dose initially improves compressive strength, owing to pore-filling and pozzolanic activity. Negative quadratic coefficients for N^2^ and T^2^ indicate that greater nano-dosages result in diminishing profits due to particle agglomeration and the inevitable strength degradation at elevated temperatures. Nano-granodiorite (NG) has the highest positive linear coefficient for N, leading to better performance at higher dosages. It also has a smaller negative coefficient for the T^2^ term, indicating better thermal stability than nano-lead (NL) and nano-magnetite (NM). The constant term, which represents baseline strength, is very consistent across all models, implying that the fundamental variance in performance originates from the materials’ individual responses to dosage and temperature, as recorded in their respective coefficients. These equations thus serve as a solid mathematical framework for predicting and optimizing the mix design of high temperature-resistant nano concrete.

CS_*NL* = 52.15381 + 2.12982*N* + 0.019277*T* − 0.341964*N*^2^ − 0.000064*T*^2^ (4)

*CS*_*NM* = 52.43839 + 2.01741*N* + 0.017046*T* − 0.187054*N*^2^ − 0.000061*T*^2^ (5)

CS_*NG* = 52.53372 + 2.59705*N* + 0.014456*T* − 0.185268*N*^2^ − 0.000057*T*^2^ (6)

#### 5.3.2. Scatter Plots for Compressive Strength of Nano Modified Concrete

**Figure 9:**
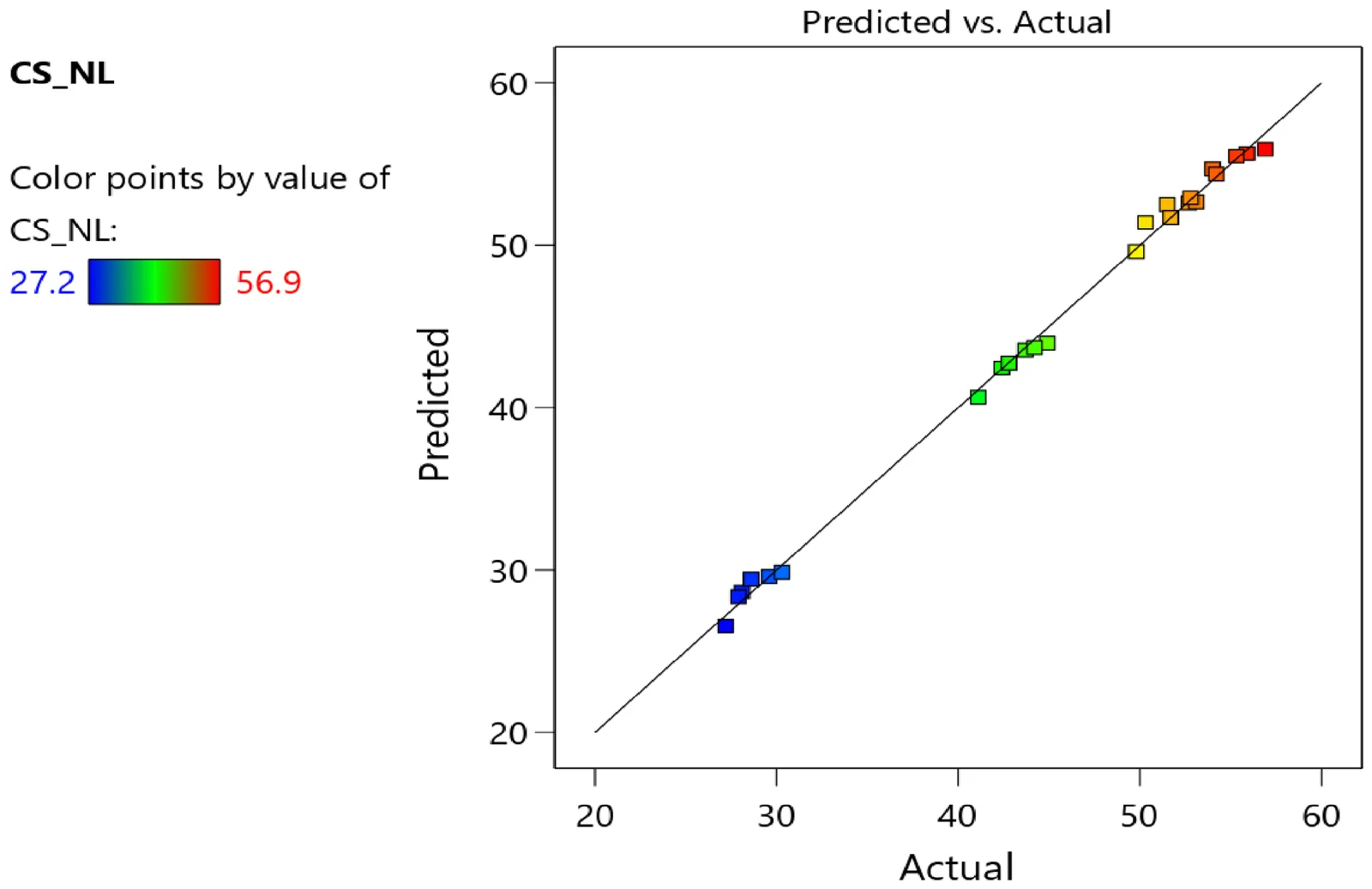
Comparison between actual and predicted values of NL-concrete compressive strength.

**Figure 10:**
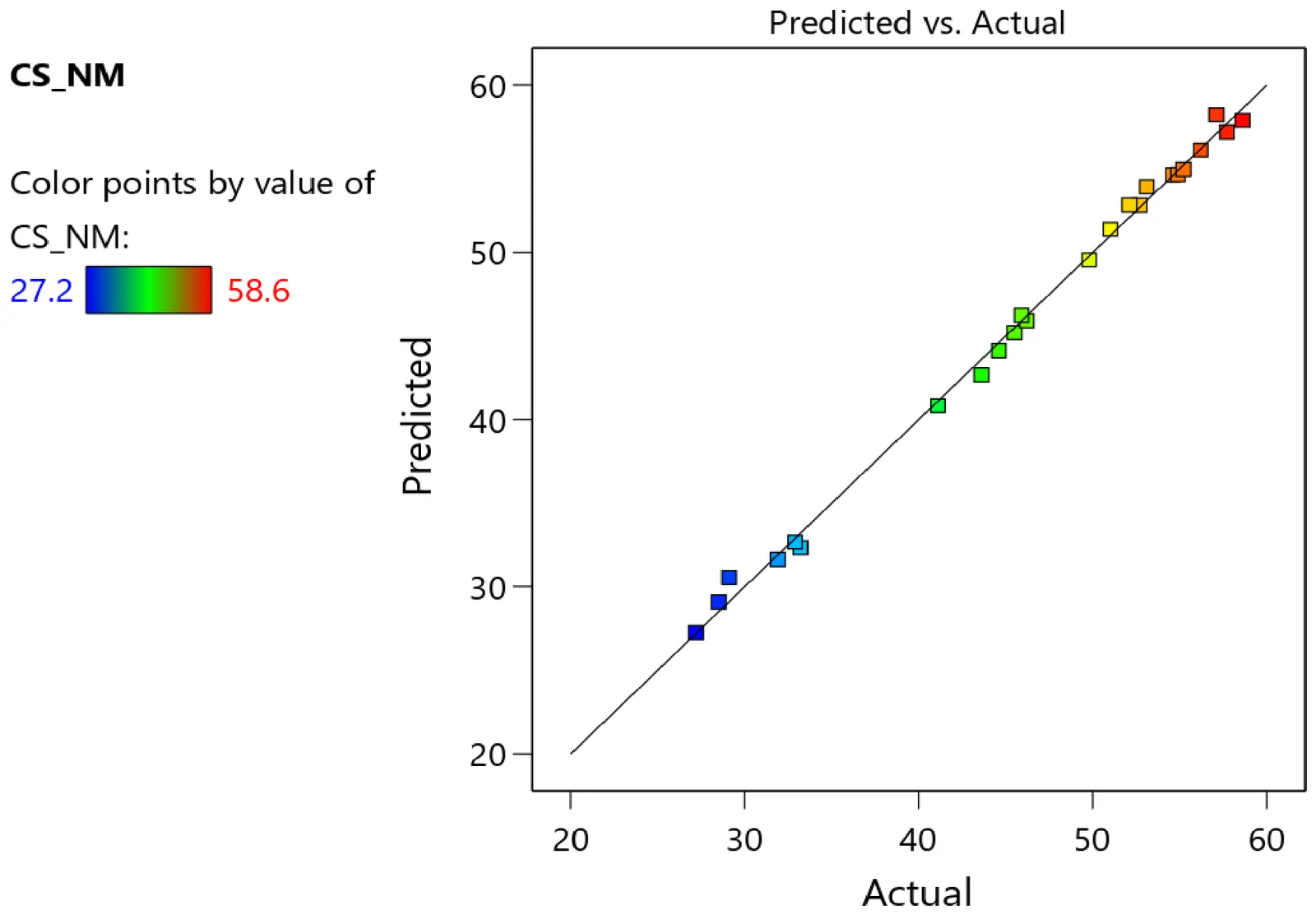
Comparison between actual and predicted values of NM-concrete compressive strength.

**Figure 11:**
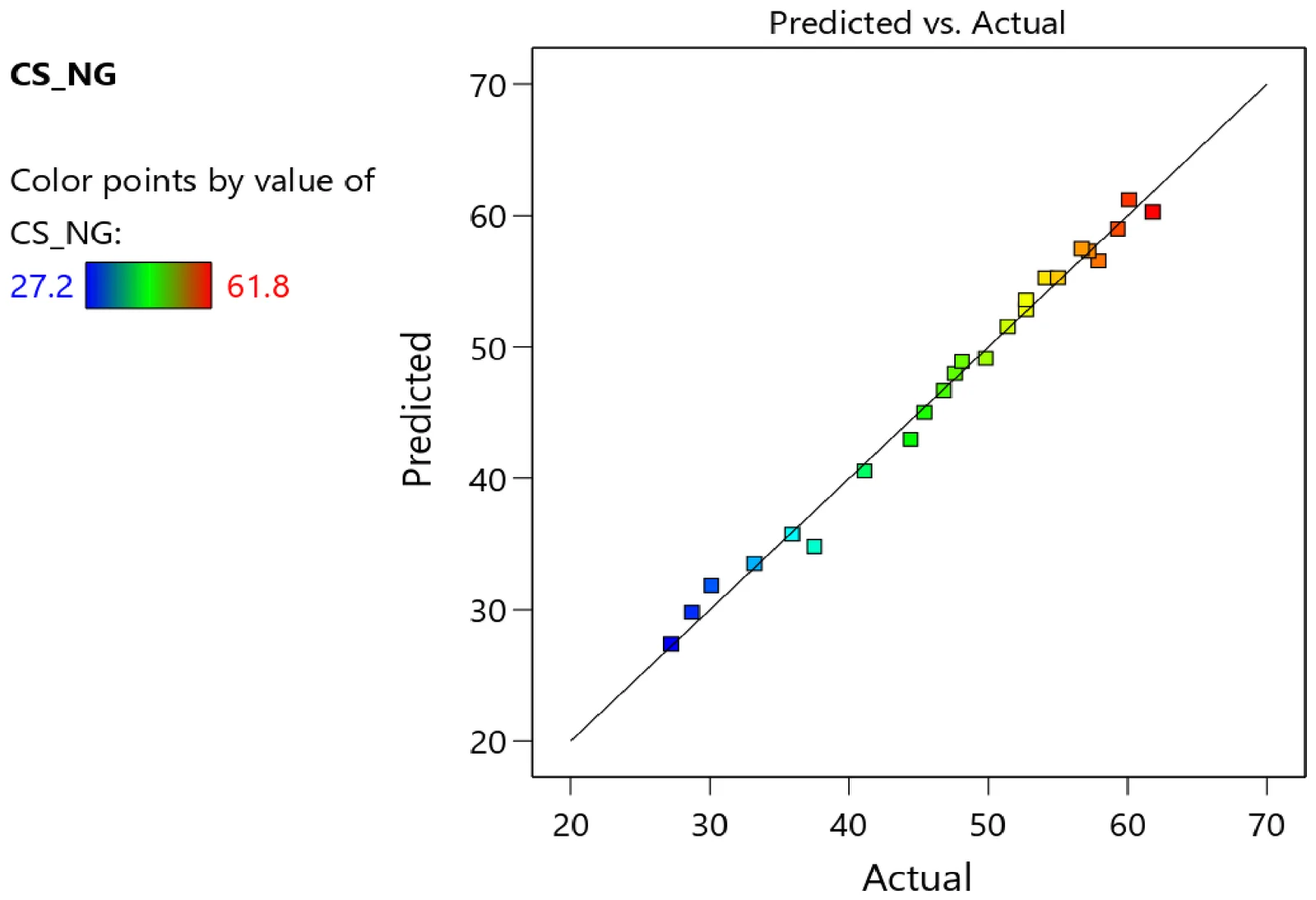
Comparison between actual and predicted values of NG-concrete compressive strength.

A primary diagnostic metric in Response Surface Methodology is the correlation plot of observed versus predicted values, which facilitates a comparative assessment of the model’s explanatory power relative to a null intercept-only model [77]. Figure 9, Figure 10 and Figure 11 show scatter plots comparing experimental and model-predicted compressive strength values, indicating a high level of predictive accuracy for the generated regression models across all three nanomaterials. The data points for nano-lead (a), nano-magnetite (b), and nano-granodiorite (c) are all closely clustered along the line of perfect agreement (y = x), with little divergence. This strong agreement implies that the mathematical models accurately represent the physical correlations between the input variables (nanomaterial dosage and exposure temperature) and the compressive strength response. The consistency of this fit across the whole data set, from lower to higher strength levels, supports the model’s robustness and lack of significant bias. The narrow dispersion of points validates the high R^2^ and adjusted R^2^ values obtained from the ANOVA. This provides strong visual evidence that the developed response surface methodology is reliable and effective for forecasting the performance of nano-engineered concrete under temperature effect.

#### 5.3.3. Diagnostic Plots for Cs

**Figure 12:**
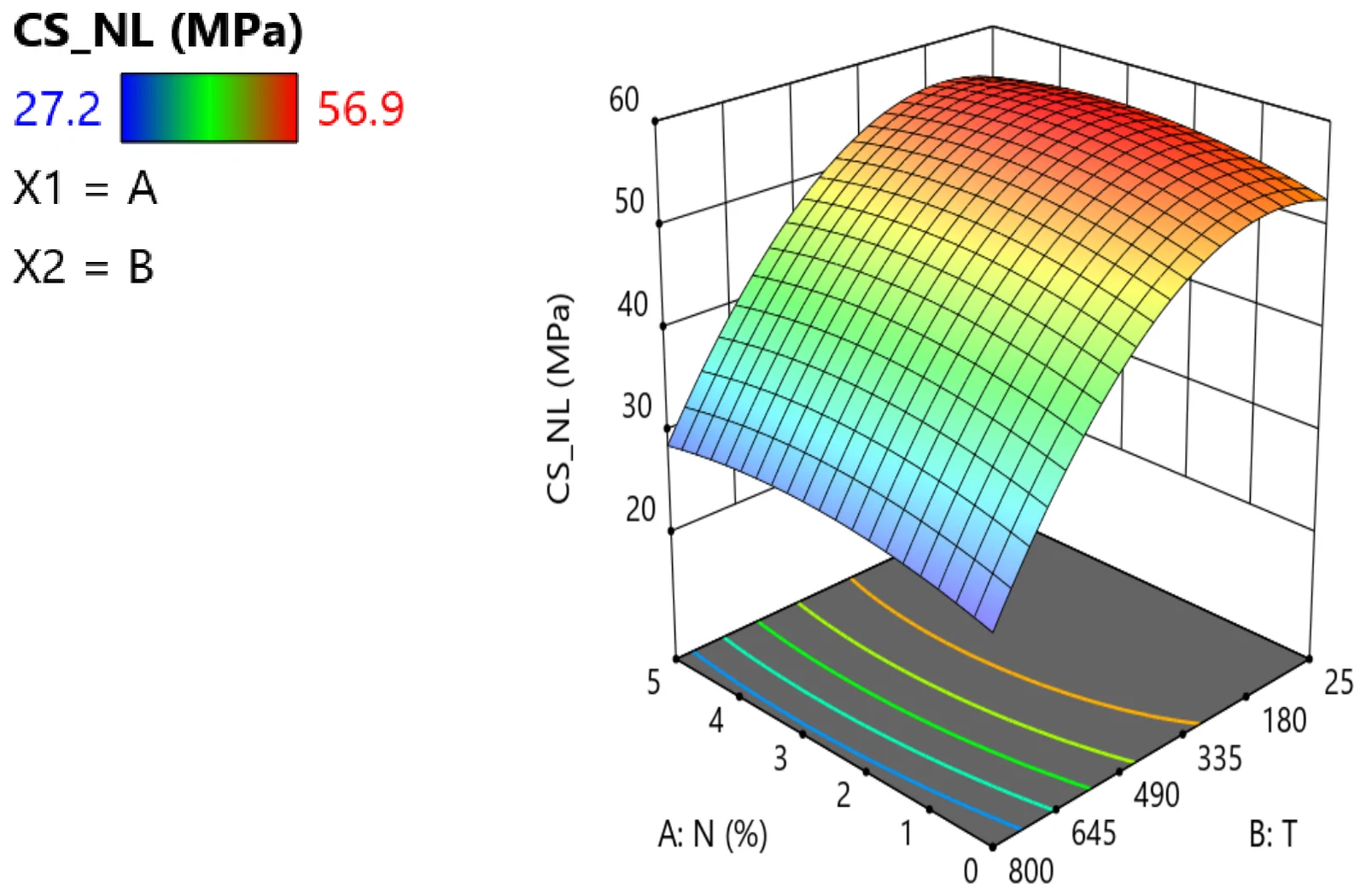
3D diagnostic plot for the effect of nano material dose and temperatures on CS of concrete modified by nano-lead oxide.

**Figure 13:**
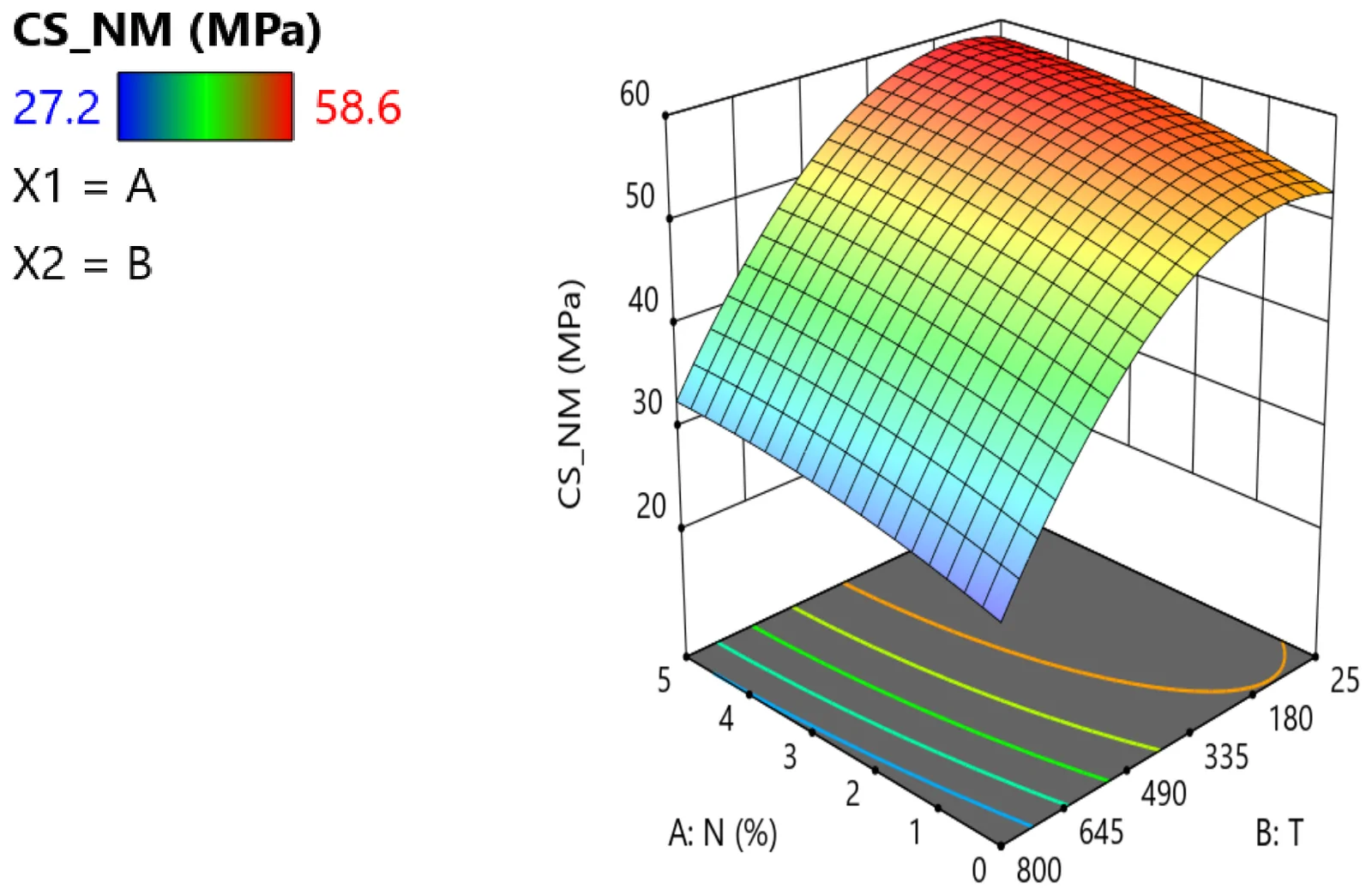
3D diagnostic plot for the effect of nano material dose and temperatures on CS of concrete modified by nano-magnetite.

**Figure 14:**
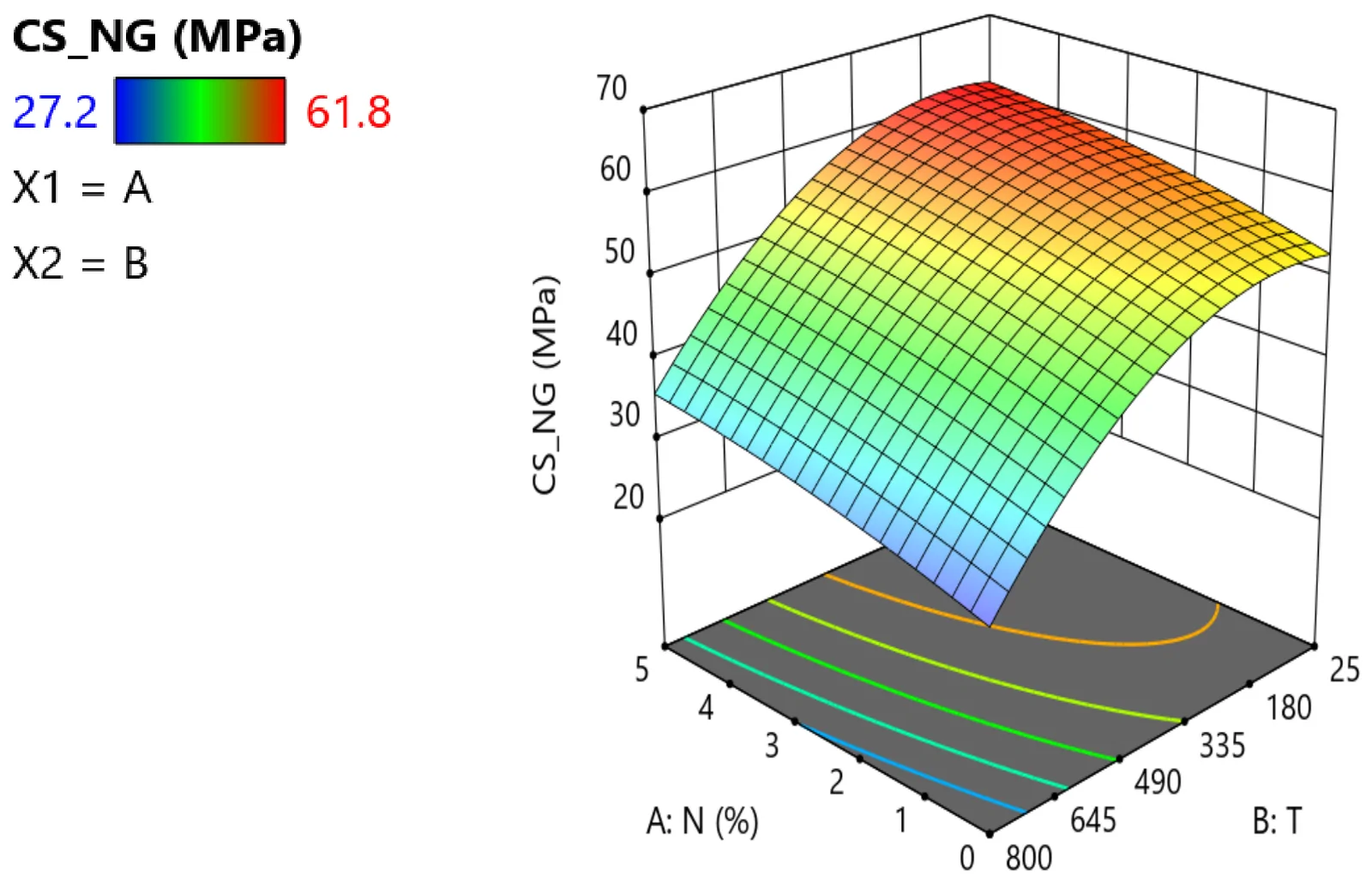
3D diagnostic plot for the effect of nano material dose and temperatures on CS of concrete modified by nano-granodiorite.

The 2D contour and 3D response surface plots in Figure 11, Figure 12 and Figure 13 visually elucidate the complex interplay between nano-material dosage (N), exposure temperature (T), and the resulting compressive strength (CS) for each modifier. A consistent trend across all three materials reveals that compressive strength degrades significantly as temperatures approach 800 °C, a behaviour characteristic of concrete’s thermo-chemical decomposition. However, the plots critically highlight how each nanomaterial mitigates this degradation differently. For nano-lead oxide (NL) and nano-magnetite (NM), the regions of highest compressive strength form distinct “ridge lines” across the response surfaces, indicating an optimal dosage range at approximately 3–4% where performance is maximized before potential agglomeration or other negative effects cause a decline (Figure 12 and Figure 13). In contrast, the surface for nano-granodiorite (NG) suggests a more robust positive relationship with dosage, with strength generally increasing up to the maximum tested level of 5% (Figure 14). Furthermore, the plots demonstrate that NG maintains superior residual strength at intermediate temperatures compared to NL and NM, as evidenced by a more elevated and less steeply sloped response surface in that region. These diagnostic plots thus provide a powerful visual confirmation that while all nanomaterials improve thermal resilience, nano-granodiorite acts as the most effective filler and pozzolanic agent, significantly preserving the concrete’s microstructure and bond integrity across a wider range of temperatures and dosages.

#### 5.3.4. Optimization of Cs by Response Surface Methodology (RSM)

**Figure 15:**
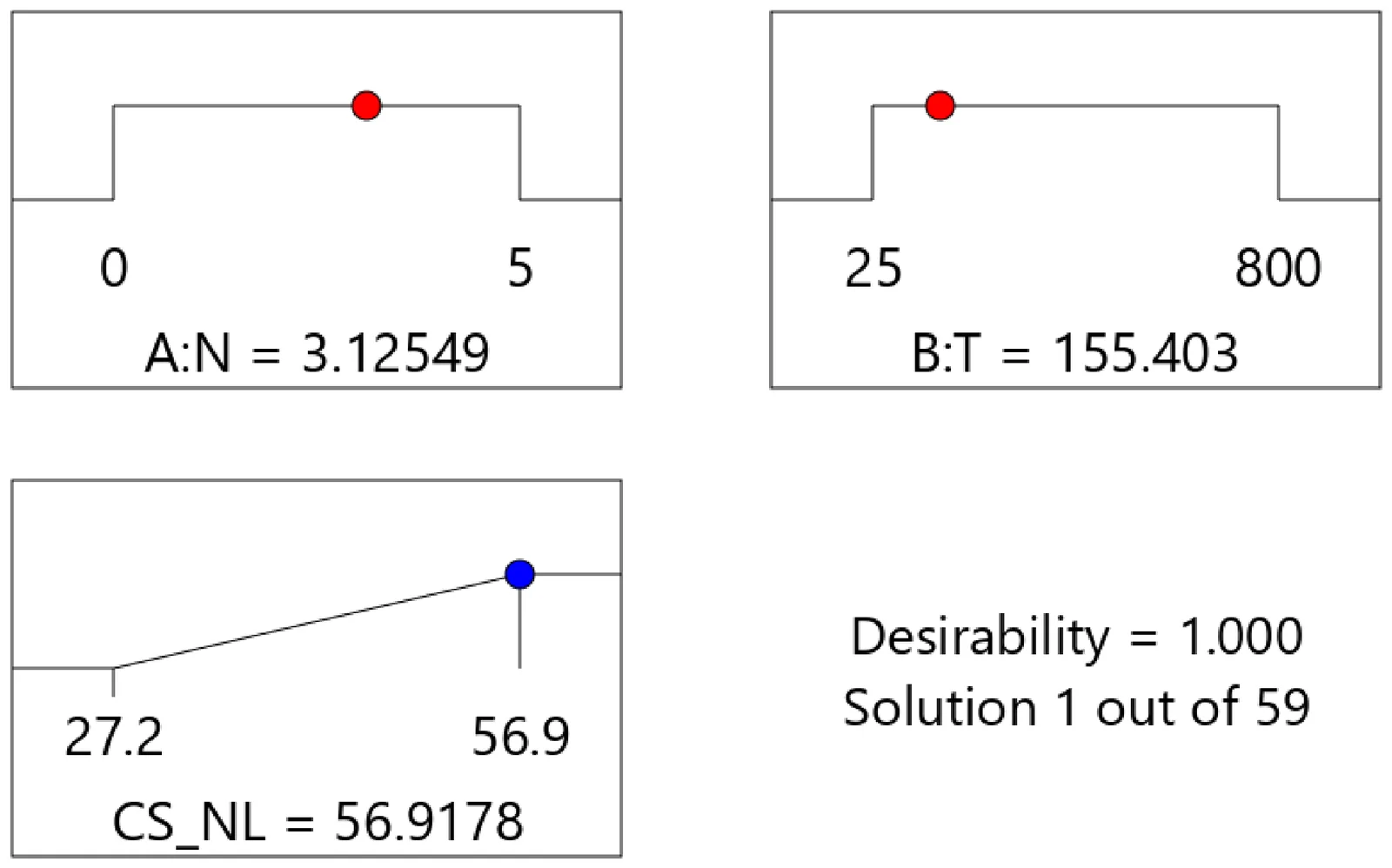
Optimization results for concrete modified by nano lead.

**Figure 16:**
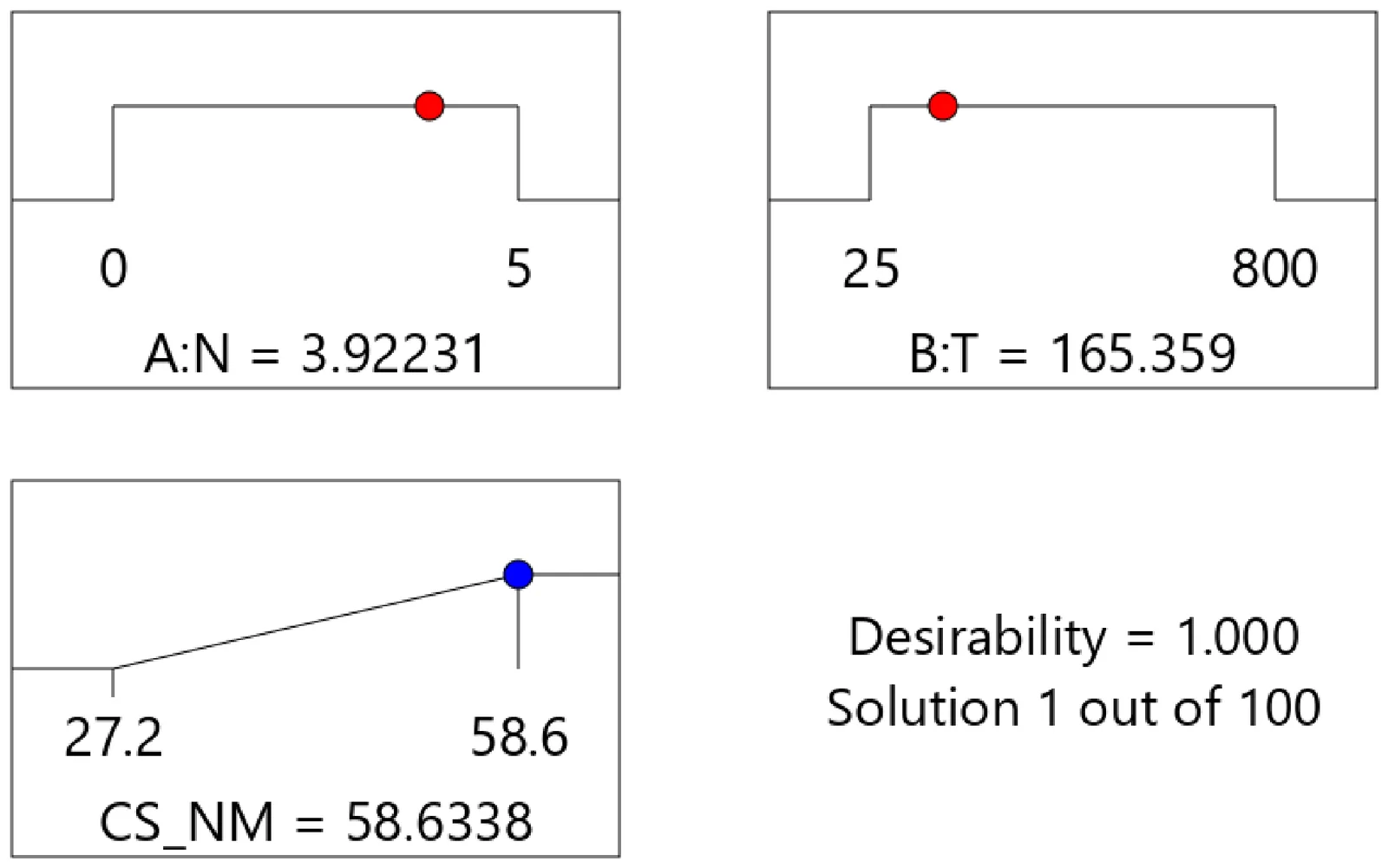
Optimization results for concrete modified by nano-magnetite.

**Figure 17:**
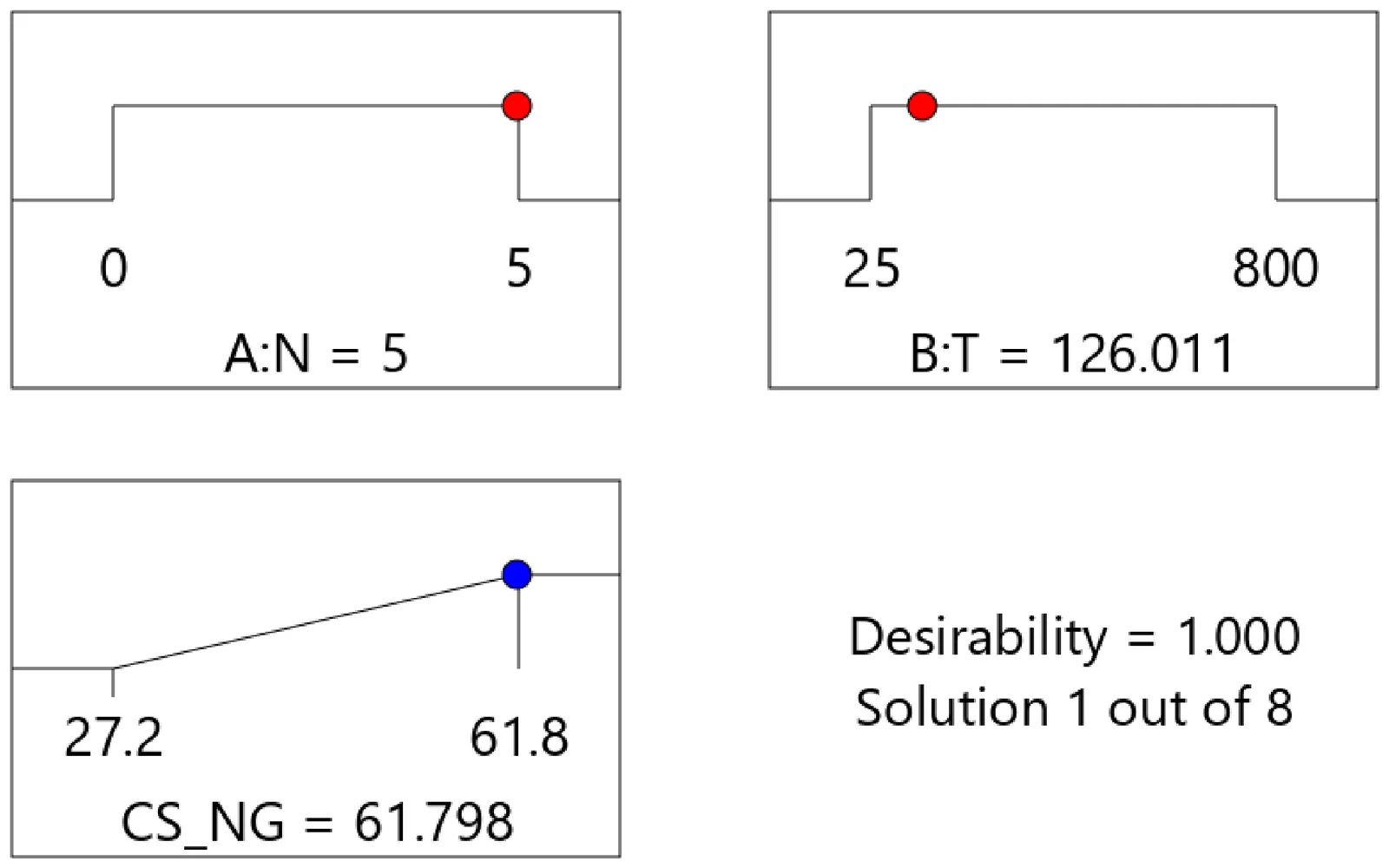
Optimization results for concrete modified by nano-granodiorite.

The numerical optimization approach effectively identified the optimal parameters to maximize compressive strength for each type of nano-modified concrete, while the nanomaterial dose (N) and temperature (T) were set within a range, with all solutions obtaining the maximum desirability of 1000. Based on the optimization results in Figure 15, Figure 16 and Figure 17, the research indicated a clear performance hierarchy among the nanomaterials. Nano-lead oxide (NL) performed best at a dosage of 3.13% and a temperature of 155.4 °C, resulting in a predicted maximum compressive strength of 56.92 MPa. To obtain its peak strength of 58.63 MPa, nano magnetite (NM) required a slightly higher dosage of 3.92% as well as a higher ideal temperature of 165.4 °C. Nano-granodiorite (NG) was the most effective additive, with the greatest anticipated compressive strength of 61.80 MPa. This higher performance was achieved at the highest possible dosage of 5%, albeit at a substantially lower optimum temperature of 126.0 °C than the other materials. These findings show that while all nanomaterials improve concrete performance, nano-granodiorite is the most effective at increasing compressive strength, especially at lower temperatures, establishing clear and specific formulation guidelines for developing high-performance, nano-engineered concrete.

## 6. Conclusions

The present study comprehensively evaluated the mechanical and thermal behavior of nano-modified concrete incorporating nano lead oxide (NL), nano magnetite (NM), and nano granodiorite (NG) as partial cement replacements at substitution levels ranging from 1% to 5% by weight of cement. Concrete specimens were subjected to elevated temperatures between 400 °C and 800 °C for an exposure duration of 120 min to assess their residual mechanical performance. By integrating experimental investigations with a statistical Response Surface Methodology (RSM) simulation, the study provided a thorough understanding of how nanomaterial type, replacement ratio, and temperature collectively influence the compressive strength of concrete exposed to thermal loading. The inclusion of nanomaterials significantly mitigates the loss of strength typically observed in conventional concrete under high-temperature conditions, thereby enhancing both its residual compressive capacity and overall structural integrity. These findings emphasize the substantial potential of nanomaterials as effective cementitious supplements for developing high-performance, thermally resistant concrete suitable for high-temperature environments. Accordingly, the key outcomes of the current study can be summarized as follows:

- At room temperatures, the inclusion of NG, NM, and NL at replacement ratios of 4%, 4%, and 3% improved the tensile strength of concrete by17.3%, 11.2%, and 8%, respectively. The inclusion of NG, NM, and NL at replacement ratios of 4%, 3%, and 3% improved the tensile strength of concrete by16.5%, 12.8%, and 9.8%, respectively.
- Nanomaterials demonstrated enhanced concrete resistance to elevated temperatures effects. Prolonged exposure to 800 °C resulted in a substantial 48.39% reduction in control mix strength due to factors including water loss, hydration product breakdown, and structural degradation.
- NG demonstrated the most promising performance in enhancing concrete’s residual compressive strength after exposure to 800 °C, with a 37.87% improvement at the optimal 4% replacement ratio. NM exhibited the second-best performance, improving in strength by 22.06% at 4%. While NL offered modest benefits, particularly a 11.4% increase at 3% replacement, it was less effective overall. NG’s superior performance is attributed to its combined void-filling and pozzolanic properties, contributing to enhanced strength and reduced microcracking.
- All three compressive strength models have high statistical significance and predictive power, as confirmed by the ANOVA results. Diagnostic plots show that NG outperforms all other nanomaterials in terms of compressive strength across a wider temperature range and dosage, followed by NM and NL.
- The numerical optimization reveals that NM is the most effective additive, with the highest compressive strength (61.80 MPa) at a 5% dosage, whereas nano-lead and NM require intermediate dosages (about 3–4%) to achieve their lower peak strength values.

Finally, it’s worth mentioning that the findings of the present study are limited by the specific experimental conditions under which the tests were conducted, including the concrete mix design, the type and percentage of cement replacement with nanomaterials, and the temperature levels and exposure durations. Therefore, generalizing these results requires further investigations involving a broader range of variables. Future studies are recommended to investigate the long-term durability of nano-modified concrete under diverse environmental and service conditions. Similar methodologies could be also employed to examine the effects of the investigated nanomaterials on the mechanical performance and durability of various concrete types, including heavyweight, lightweight, ultra-high-performance, and geopolymer concrete. Further research should also evaluate the environmental implications of incorporating lead-based nanomaterials, including their potential leaching during service and after deterioration, and assess appropriate encapsulation measures to minimize associated risks. In addition, comprehensive microstructural characterization using complementary techniques, such as scanning electron microscopy (SEM), energy-dispersive X-ray spectroscopy (EDS), X-ray diffraction (XRD), and mercury intrusion porosimetry (MIP), would provide deeper insights into the mechanisms responsible for microstructural changes and residual mechanical performance after heating. Furthermore, Artificial intelligence techniques, including artificial neural networks, ensemble learning, and hybrid optimization algorithms, could also be employed to predict residual mechanical properties as functions of temperature exposure, nanomaterial type and dosage, and other relevant mixture parameters. Integrating experimental results with microstructural characteristics could further improve predictive accuracy, identify optimal mixture proportions, and support the development of nano-modified concrete with enhanced resistance to elevated temperatures.

## Figures and Tables

**Table 1:** Chemical analysis of utilized powders.

	OPC	NS	NL	NM	NG
SiO_2_	20.55	99.86	-	0.02	64.10
CaO	60.65	0.03	-	-	5.30
Al_2_O_3_	3.90	0.02	-	-	16.32
Fe_2_O_3_	2.33	0.01	-	99.42	5.15
MgO	1.72	0.01	-	-	1.35
SO_3_	2.52	-	-	-	0.09
Na_2_O	0.45	-	-	-	3.41
K_2_O	0.17	-	-	-	0.12
PbO	-	-	99.50	-	-
P_2_O_5_	0.21	-	-	-	0.32
TiO_2_	0.37	0.01	-	0.03	0.78
LOI ^∗^	3.86	0.05	0.05	0.10	0.90

LOI ^∗^: Loss on ignition at 1000 °C.

**Table 2:** Concrete ingredients (kg/m^3^).

Mix	Cement	NS	Nano Additives		
			NL	NM	NG
Control (CO)	500	10	----	----	----
1NL	495	10	5	----	----
2NL	490	10	10	----	----
3NL	485	10	15	----	----
4NL	480	10	20	----	----
5NL	475	10	25	----	----
1NM	495	10	----	5	----
2NM	490	10	----	10	----
3NM	485	10	----	15	----
4NM	480	10	----	20	----
5NM	475	10	----	25	----
1NG	495	10	----	----	5
2NG	490	10	----	----	10
3NG	485	10	----	----	15
4NG	480	10	----	----	20
5NG	475	10	----	----	25

----: Refers to zero addition of the nanomaterial.

**Table 3:** Compressive strength results after various temperature degrees exposure.

Temp. (°C)	CO	Nano Lead Oxide					Nano Magnetite					Nano Granodiorite				
		1%	2%	3%	4%	5%	1%	2%	3%	4%	5%	1%	2%	3%	4%	5%
25	52.70	54.20	55.30	56.90	55.90	54.00	54.90	56.20	57.70	58.60	57.10	55.00	57.20	59.30	61.80	60.10
400	49.80	50.30	51.50	52.80	53.10	51.70	51.00	52.10	53.10	54.60	55.20	51.40	52.70	54.10	57.90	56.70
600	41.10	42.40	43.70	44.90	44.20	42.80	43.60	44.60	45.50	46.20	45.90	44.40	45.40	46.80	47.60	48.10
800	27.20	27.90	28.60	30.30	29.60	28.10	28.50	29.10	31.90	33.20	32.90	28.70	30.10	33.20	37.50	35.90

**Table 4:** ANOVA for compressive strength responses.

Nano Material	Response	F-Value	*p* Value	Adequate Precision	R^2^	Adjusted R^2^	Predicted R^2^	C.V. %
**Nano lead oxide**	CS_NL	1626.13	<0.0001	103.934	0.9971	0.9965	0.9954	1.39
**Nano magnetite**	CS_NM	1378.72	<0.0001	102.128	0.9966	0.9958	0.9945	1.45
**Nano granodiorite**	CS_NG	501.32	<0.0001	66.3469	0.9906	0.9886	0.9855	2.36

## Data Availability

The original contributions presented in this study are included in the article. Further inquiries can be directed to the corresponding author.
